# Flower-like NiAl-LDH/BiVO_4_ Z-scheme photocatalysts for sunlight-driven degradation of azo dye: performance and mechanistic insights

**DOI:** 10.1039/d5ra06146f

**Published:** 2025-10-07

**Authors:** Manpreet Kaur, Pritam Hait, Soumen Basu

**Affiliations:** a Department of Chemistry and Biochemistry, Thapar Institute of Engineering and Technology (TIET) Patiala-147004 Punjab India soumen.basu@thapar.edu; b TIET-Virginia Tech Center of Excellence in Emerging Materials, Thapar Institute of Engineering and Technology (TIET) Patiala-147004 Punjab India

## Abstract

Layered double hydroxide (LDH)-based materials have garnered significant attention as versatile photocatalysts for environmental remediation, particularly for the abatement of dye-laden wastewater, owing to their structural tunability, chemical robustness, and facile synthetic routes. In this context, a series of NiAl-LDH/BiVO_4_ (NAL/BV) Z-scheme heterojunction nanocomposites were constructed by loading 5–15% (wt%) of BiVO_4_ onto LDH *via* an *ex situ* fabrication method, and evaluated for photocatalytic degradation of Congo red (CR), a typical azo dye, under solar irradiation. The structural, morphological, and optical attributes of the nanocomposites were meticulously elucidated through comprehensive analyses, including XPS, FTIR, PL, UV-DRS, FESEM, HRTEM, and BET surface area measurements. The optimized 5-NAL/BV composite exhibited a flower-like morphology with an augmented surface area, promoting efficient charge separation and enhanced photocatalytic activity. At a catalyst loading of 0.3 g L^−1^, it achieved 94.3% CR degradation within 2 hours, with an apparent kinetic rate constant of 0.01673 min^−1^ and a synergy factor of 5.67. The effects of contaminant concentration, catalyst dose, pH, and light source on activity were systematically studied. TOC analysis confirmed 50% mineralization, while scavenging studies identified superoxide radicals as the primary reactive species. HRMS analysis elucidated degradation intermediates, and post-cycle characterization confirmed structural stability over six cycles. Moreover, a comparative analysis with previously reported studies demonstrates that this hybrid acts as a superior photocatalyst for the decomposition of hazardous dyes, highlighting the potential of NAL/BV nanocomposites for solar-driven wastewater treatment and environmental remediation.

## Abstract 2

LDH-based materials are promising photocatalysts for environmental remediation, especially dye-laden wastewater treatment, due to their structural tunability, chemical stability, and simple synthesis.

## Graphical abstract

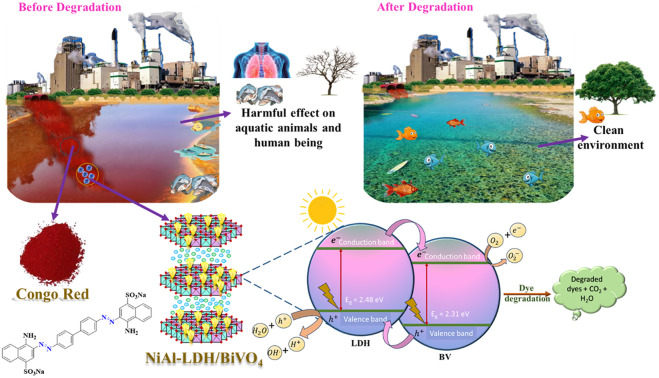

## 1. Introduction

Dyes are chemically defined as aromatic organic molecules primarily utilized to impart color to diverse substrates such as paper, hair, textile fibres, foodstuffs, and more, for aesthetic purposes.^1^ However, inefficiencies inherent to conventional dyeing processes result in substantial dye losses (20–50%), with residual fractions discharged into aquatic systems. Such effluents impose severe ecological and health hazards owing to their toxicity, recalcitrance, and aesthetic impacts.^2,3^ Global dye production exceeds 700 000 tonnes annually, and a considerable fraction ultimately enters wastewater streams.^4^ Certain dyes, especially azo dyes, exhibit high resistance to degradation owing to their complex aromatic systems and persistent environmental behavior. Consequently, the remediation of dye-laden wastewater is viewed as a major environmental challenge, requiring urgent attention and innovative solutions.^5,6^ A wide repertoire of treatment technologies has been explored, including coagulation–flocculation, biological degradation, electrochemical oxidation, ozonation, membrane filtration, adsorption, and photocatalysis.^7^ Of these, heterogeneous photocatalysis is particularly compelling, as it operates under mild conditions, efficiently degrades toxic pollutants into benign end products (H_2_O and CO_2_), and poses minimal risk of secondary pollution.^8^ Classical photocatalysts such as TiO_2_, CdS, SrTiO_3_, ZnO, *etc.*, have been widely investigated owing to their chemical stability, environmental compatibility, and cost effectiveness. Nonetheless, their intrinsic limitations, such as wide band gaps that confine activity to the UV region and ultrafast electron–hole recombination, continue to hinder practical application. Despite extensive endeavours in surface modification and band-structure engineering, these challenges remain unresolved, driving the pursuit of visible-light-responsive semiconductors with improved charge separation.^9–15^ In this regard, layered double hydroxide (LDH)-based nanocomposites are particularly promising due to their two-dimensional structure, visible–light activity, surface basicity, and highly tunable composition.^16,17^

LDH represent a class of ceramic materials characterized by the general formula [M_1−*x*_^2+^M_*x*_^3+^(OH)_2_]^*x*+^[*A*^*n*−^]_*x*/*n*_·*y*H_2_O. Here, divalent (M^2+^) and trivalent (M^3+^) metal cations are octahedrally coordinated with hydroxide ions to form positively charged brucite-like layers, electrostatically neutralized by exchangeable interlayer anions (OH^−^, CO_3_^2−^, NO_3_^−^, *etc.*). These intercalated anions impart structural integrity, ion-exchange capability and chemical tunability, rendering LDHs attractive for catalysis, adsorption, and environmental remediation.^18,19^ The versatility of the cationic matrix and interlayer anionic framework in LDHs plays a crucial role in enhancing their photocatalytic performance.^20^ Among them, NiAl-LDH has received particular attention as an *n*-type semiconductor exhibiting morphologies such as nanosheets and hierarchical microspheres that facilitate co-catalyst support and photocarrier migration. Its photocatalytic activity can be enhanced by adjusting the Ni/Al composition during synthesis and modifying their physicochemical properties.^21^ Unfortunately, poor intrinsic conductivity and irreversible nanosheet restacking hinder charge transport and ultimately lead to a reduction in the photocatalytic efficiency.^22–24^ To mitigate these issues, strategies such as integration with conductive materials, plasmonic metal loading, metal doping, and heterojunction formation have been explored.^25^ Notably, incorporating metal oxides to form heterojunctions is especially effective, as this approach not only extends the absorption of visible light but also induces synergistic interactions that enhance charge carrier separation, reduce recombination rates, and facilitate efficient charge transfer across interfaces, resulting in a significant improvement in photocatalytic efficiency.^26^

Among various metal oxides (WO_3_, Fe_3_O_4_, NiO, *etc.*), bismuth vanadate (BiVO_4_) stands out due to its exceptional electronic and structural characteristics, positioning it as a promising photocatalyst. Its pronounced absorption in the visible region, stemming from a band gap of ∼2.4 eV, enables effective pollutant degradation, while a suitably aligned valence band (∼2.6 eV *vs.* NHE) promotes the generation of oxidative charge carriers.^27^ Additionally, BiVO_4_ exhibits chemical stability across pH 3–11 and, due to its facile synthesis, low cost, and environmentally benign nature, represents a promising photocatalyst for visible-light-driven applications.^27,28^

Previous reports have demonstrated the photocatalytic potential of both NiAl-LDH and BiVO_4_-based composites for pollutant degradation. For instance, Salehi *et al.* achieved 93% degradation of methyl orange and rhodamine B using g-C_3_N_4_/NiAl-LDH.^29^ Chen *et al.* reported a BiVO_4_/BaSnO_3_ p–*n* heterojunction photocatalyst supported on Halloysite nanotubes, achieving 94.1% methylene blue degradation under visible light.^30^ Abou Taleb synthesized an MXene-based ternary nanocomposite comprising BiVO_4_ and Bi_2_S_3_ for industrial wastewater treatment.^31^ Debapriya Pradhan *et al.* fabricated a Ce-doped Co_3_O_4_/BiVO_4_ heterojunction using Mangifera indica leaf extract for efficient Congo red degradation and supercapacitor applications.^32^ Siregar *et al.* synthesized Ni/Al LDH anchored on biochar and graphite for efficient Congo red adsorption.^33^ Despite these advances, the solar-driven degradation of Congo red using NiAl-LDH/BiVO_4_ heterojunctions with different weight ratios remains unexplored. Moreover, the mechanistic pathway—including identification of intermediates and final products—has yet to be elucidated, which is critical for evaluating photocatalytic efficiency and environmental sustainability. Although Luo *et al.* fabricated a BiVO_4_/NiAl-LDH heterojunction for the degradation of tetracycline, their study did not systematically examine catalyst loading, initial pollutant concentration, or benchmark comparisons with TiO_2_-P25.^34^

To address these gaps, NiAl-LDH/BiVO_4_ nanocomposites with varied weight percentages were synthesized through an *ex situ* approach and characterized using XRD, XPS, PL, FESEM, BET, and related techniques. The effectiveness of the fabricated composites was assessed through the solar-induced degradation of CR. Critical operational parameters (pollutant concentration, photocatalyst dosage, and solution pH) were meticulously investigated to determine optimum conditions. Kinetics, synergy factor, and the underlying photocatalytic mechanism of NiAl-LDH/BiVO_4_ were comprehensively examined and critically discussed based on the experimental findings. Scavenging experiments were conducted to identify the reactive species involved in the decomposition process, while cyclic degradation studies were performed to evaluate the stability and reusability of the photocatalyst. High-resolution mass spectrometry (HRMS) was employed to identify intermediate species and final degradation products, enabling the proposal of a plausible degradation pathway.

## 2. Materials and methods

### 2.1 Materials

Materials used in the synthesis are available in SI.

### 2.2 Synthesis of LDH

To synthesize NiAl-LDH *via* a hydrothermal route, an aqueous solution containing 0.87 g of Ni(NO_3_)_2_·6H_2_O and 0.375 g of Al(NO_3_)_3_·9H_2_O was magnetically agitated for 15 minutes. Subsequently, 0.592 g of NH_4_F and 2.40 g of urea were added, and the mixture was further stirred for 30 minutes to ensure homogeneity. The resulting solution was transferred into a 100 mL Teflon-lined stainless-steel autoclave and subjected to thermal treatment at 120 °C for 24 hours. Upon natural cooling to room temperature, the precipitate was collected *via* centrifugation, washed thoroughly with distilled water and absolute ethanol, and finally oven-dried at 60 °C for 12 hours.^35^

### 2.3 Synthesis of BiVO_4_ powder

Bi(NO_3_)_3_·5H_2_O (5 mmol) and NH_4_VO_3_ (5 mmol) were separately dissolved in 10 mL of 4 M HNO_3_ and 2 M NaOH solution, respectively. After 30 minutes of vigorous stirring, 250 mg of sodium dodecyl benzene sulphonate (C_18_H_29_NaO_3_S) was added to both solutions. The resulting solutions were then mixed, and the acidity was neutralized using 2 M NaOH while stirring continuously. The resulting solution was transferred to a 100 mL Teflon-lined stainless-steel autoclave and heated to 200 °C for 1.5 hours. Upon completion, the precipitates were thoroughly washed several times with double-distilled water, followed by drying in a hot air oven at 60 °C. The end result was a yellowish BiVO_4_ powder, referred to as BV.^36,37^

### 2.4 Preparation of NiAl-LDH/BiVO_4_ hybrids

**Scheme 1:**
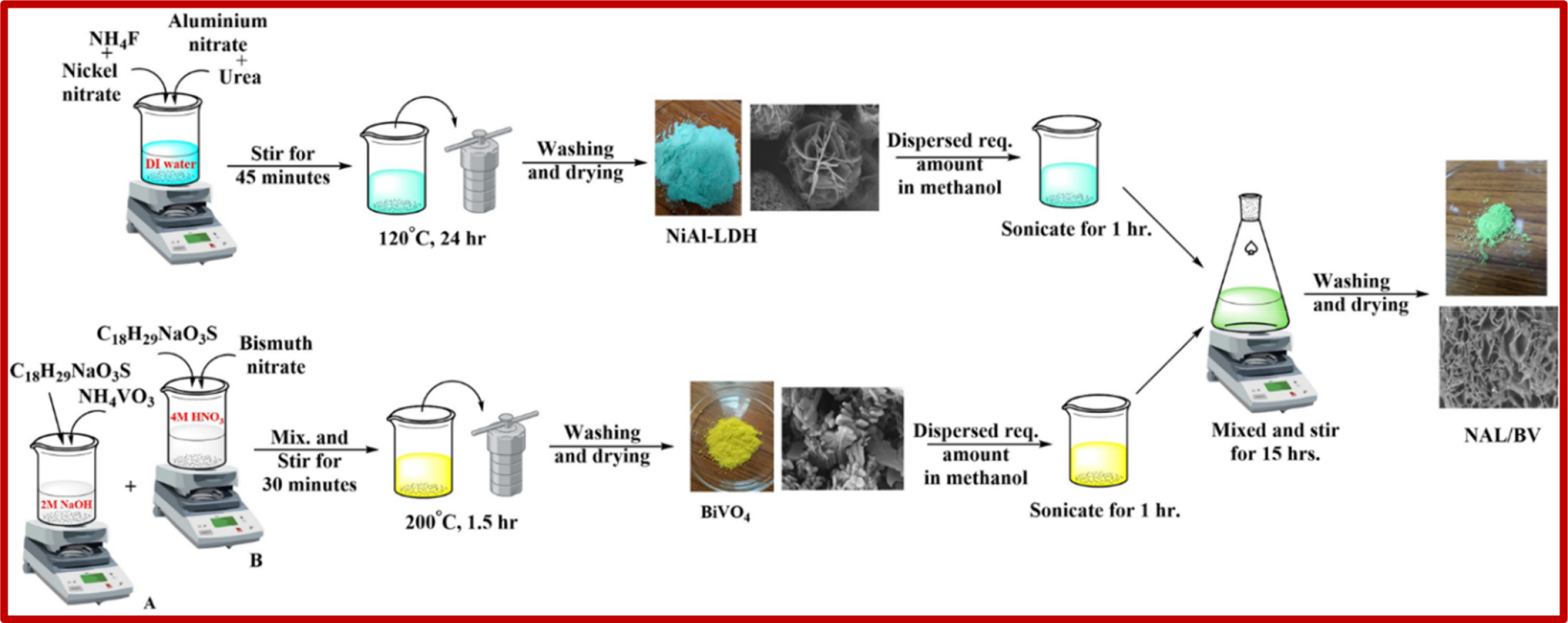
Synthesis pathway of different NAL/BV photocatalysts.

The NiAl-LDH/BiVO_4_ composites were synthesized *via* electrostatic assembly, with varying weight percentages of BiVO_4_, as shown in Scheme 1. A measured amount of NiAl-LDH was dispersed in ethanol using sonication for 60 minutes, and a specified quantity of BV was similarly dispersed in ethanol. Afterwards, the two suspensions were combined and stirred for 15 hours at 600 rpm. After centrifuging, the obtained material was subjected to drying at 60 °C in a hot air oven. The prepared NiAl-LDH/BiVO_4_ composites, containing 5, 10, and 15 wt% of BV, were labeled as 5-NAL/BV, 10-NAL/BV, and 15-NAL/BV, respectively.

### 2.5 Fabrication of working electrode

Working electrodes for EIS measurements were prepared by dispersing 3 mg of catalyst in 100 μL deionized water, 20 μL Nafion solution (binder), and 180 μL ethanol, followed by ultrasonication to obtain a homogeneous suspension. The suspension was drop-cast onto pretreated Ni foam (0.5 × 1.0 cm), and the electrodes were dried at room temperature for 24 h.

### 2.6 Characterization methods

Comprehensive details regarding the characterization techniques and instruments employed are provided in SI(S2).

### 2.7 Photocatalytic studies

The photocatalytic potential of the synthesized nanocomposites was assessed by degrading CR, an organic dye, under catalytic conditions. Prior to irradiation, 10 mL of 25 ppm CR solution containing 0.3 g L^−1^ catalyst was magnetically agitated in the absence of light for 90 minutes to ensure equilibrium between adsorption and desorption. Afterwards, the reaction mixture was exposed to natural sunlight for 120 minutes. The experimental study was carried out over August and September, 2024, during which the LICOR pyranometer recorded solar irradiance values between 700 and 880 W m^−2^, reflecting consistent light conditions. Each trial was performed in triplicate, and the resulting data are presented with error bars, accounting for a measurement uncertainty of 5%. Aliquots of 5 mL were periodically extracted from the reaction system, centrifuged at 5000 rpm for 4 minutes to separate the catalyst, and the supernatant was examined at 498 nm using a UV-visible spectrophotometer to quantify the remaining CR concentration. The degradation efficiency (% Deg) of CR was determined using eqn (1).1
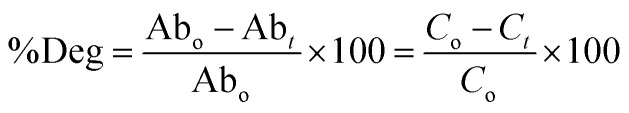
Here, Ab_o_ and Ab_*t*_ indicate the absorbance of CR at time zero and at time *t*, respectively, while *C*_o_ and *C*_*t*_ correspond to the initial and remaining concentrations of the dye.^38^ The reusability of the optimal photocatalyst was assessed by subjecting the material to successive cycles, wherein it was recovered through washing, centrifugation, and drying after each run. Total organic carbon analysis was carried out to examine the effectiveness of the photocatalytic treatment in degrading CR. To calculate the % TOC eradication, eqn (2) was used.2
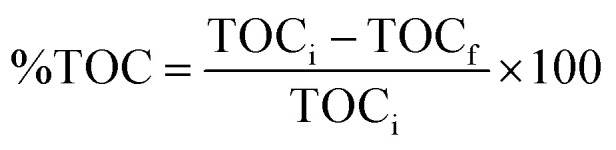
where TOC_i_ and TOC_f_ represent the total organic carbon content at the beginning and end of the photocatalytic process, respectively.^39^

## 3. Results and discussion

### 3.1 Spectral analysis

**Fig. 1:**
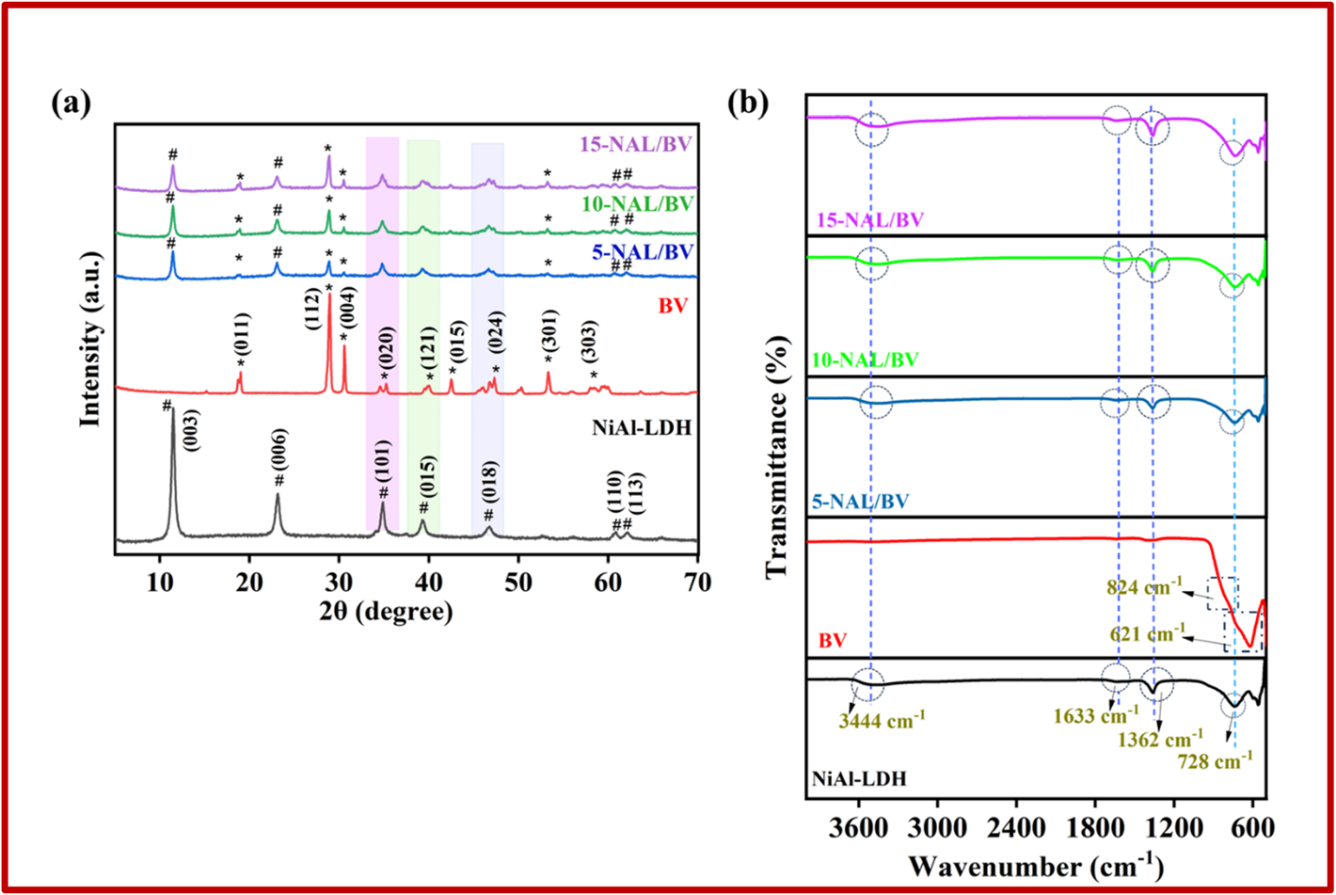
(a) Powdered XRD, and (b) FTIR plots of NiAl-LDH, BiVO_4,_ and LDH/BV composites.

X-ray diffraction pattern was utilized to analyze the crystallographic phase, structural properties, and purity of the synthesized NiAl-LDH, BV, and their hybrid composites. As shown in Fig. 1(a), the XRD peaks at 2*θ* = 11.48°, 23.16°, 34.88°, 39.38°, 46.78°, 60.88°, 62.16° corresponds to d(003), d(006), d(101), d(015), d(018), d(110), and d(113) crystallographic planes, respectively, confirming the formation of hexagonal NiAl-LDH (JCPDS file no: 15-0087).^40^ The XRD analysis of BV revealed peaks at 18.74° (011), 28.96° (112), 30.58° (004), 34.62° (200), 35.24° (020), 39.82° (211), 40.04° (121), 42.5° (015), 46.06° (123), 46.8° (204), 47.32° (024), 50.32° (220), 53.32° (301) and 58.46° (303) (JCPDS file no: 83-1699), confirms the successful formation of hexagonal BiVO_4_.^41^ In the *x*-NAL/BV composites (*x* = 5%, 10%, 15%), the XRD patterns exhibited reflections from both NiAl-LDH and BV, accompanied by slight shifts and partial overlaps. These shifts can be attributed to lattice distortion and interfacial strain, indicating a strong structural coupling between NiAl-LDH and BV. In contrast, a simple physical mixture would only exhibit an overlay of the two parent phases without any positional changes. Therefore, the observed diffraction shifts confirm the successful formation of the NAL/BV composite rather than mere physical mixing. Moreover, the relative intensity of BV peaks increased with higher BV loading (5–15%), demonstrating the effective incorporation of BV into the composite matrix, while the absence of extraneous peaks attests to the high phase purity of the synthesized samples. These interfacial structural modifications are expected to enhance charge transfer and suppress electron–hole recombination, and improve the photocatalytic activity of the composite.

Fig. 1(b) illustrates the FTIR spectra of NiAl-LDH, BV, and *x*-NAL/BV hybrids. In the NiAl-LDH spectrum, absorption bands appeared at 3444 cm^−1^ and 1633 cm^−1^ corresponding to the stretching and bending vibration of O–H bonds, respectively. A prominent peak at 1362 cm^−1^ is ascribed to the bending vibrations of CO_3_^2−^ ions incorporated within the layered matrix, whilst the peaks appeared in the lower fingerprint region (below 800 cm^−1^) are assigned to translational modes of metal–oxygen–metal (Al–O–Ni) and metal–oxygen (Al–O, Ni–O) linkages.^42,43^ In case of BV, a broad and intense band with poorly resolved shoulders is evident, encompassing peaks at 824 cm^−1^ and 621 cm^−1^, which are associated with the stretching (symmetric and asymmetric) and bending modes of V–O bonds.^44,45^ It is noteworthy that the NAL/BV composites preserve all the characteristic peaks of LDH with slight shifting in the wavenumber due to bismuth vanadate loading, providing clear evidence for the successful preparation of the nanocomposites.

**Fig. 2:**
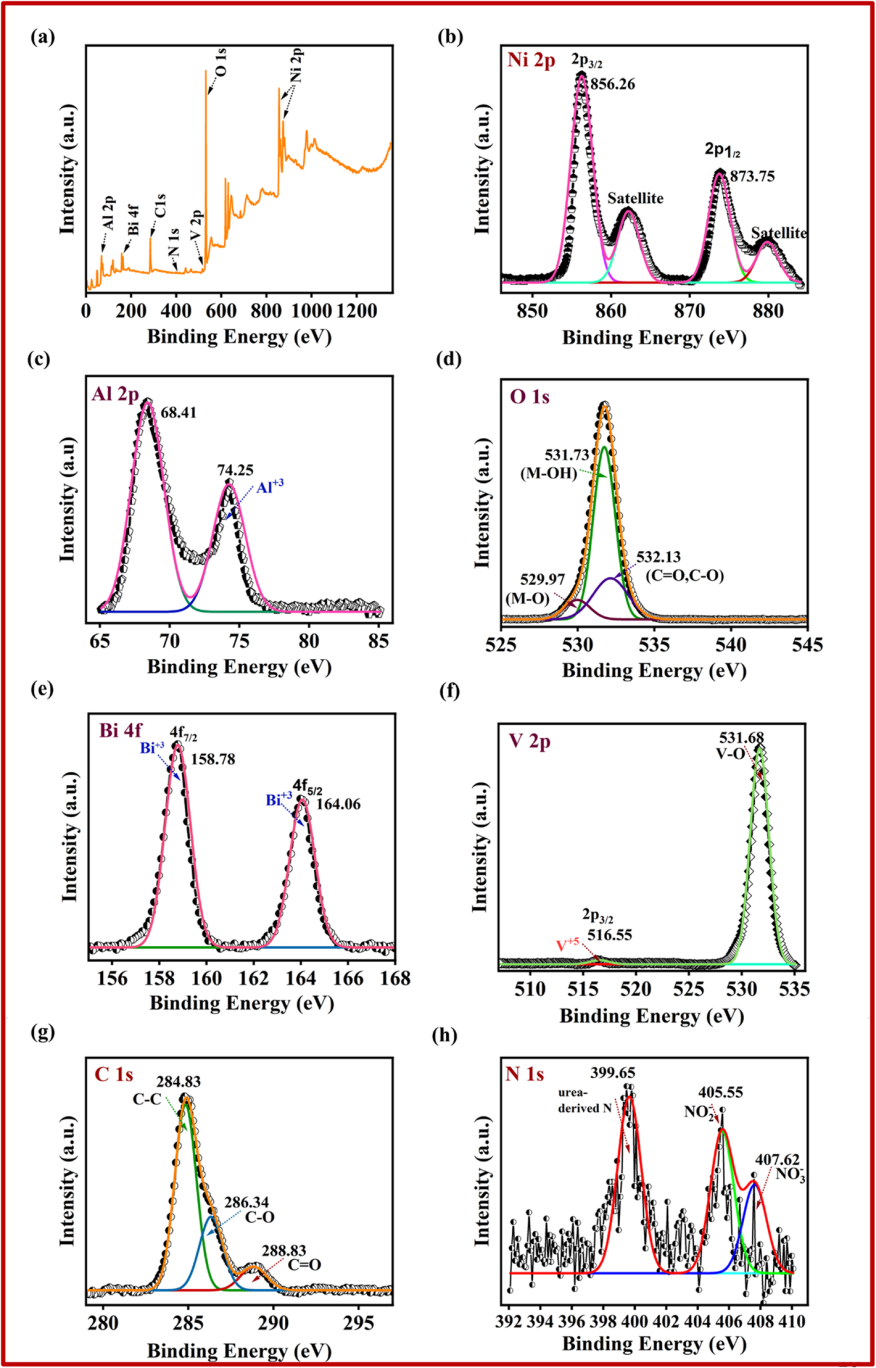
XPS spectrum of 5-NAL/BV composite; (a) elemental survey scan, (b) Ni 2p, (c) Al 2p, (d) O 1s, (e) Bi 4f, (f) V 2p, (g) C 1s, and (h) N 1s.

XPS analysis was done to determine the elemental composition, core-level binding energies, and chemical states of the constituent elements. This technique also allows differentiation of spin–orbit splitting in metal ions, reflected by distinct binding energies. The full-range XPS spectrum of the 5-NAL/BV composite, as depicted in Fig. 2(a), reveals the presence of Ni, Al, Bi, V, C, O, and N, each identified by their respective binding energies. Peak deconvolution was performed using a least-squares Gaussian fitting model. The Ni 2p spectrum in Fig. 2(b) reveals two prominent peaks at 856.26 and 873.75 eV, corresponding to the Ni 2p_3/2_ and Ni 2p_1/2_ components, respectively, which are indicative of the Ni^2+^ oxidation state. The presence of satellite peaks further confirms the high-spin configuration of divalent nickel in the sample.^46,47^ Similarly, the Al 2p spectrum (Fig. 2(c)) exhibits peaks at 68.41 and 74.25 eV, which are indicative of Al^3+^ species, thereby confirming the trivalent state of aluminium within the composite.^48,49^ The O 1s spectrum (Fig. 2(d)) presents three notable peaks at 529.97, 531.73, and 532.13 eV, which are attributed to vanadium–oxygen bonds, surface hydroxyl groups associated with the NiAl-LDH layers, and carbonyl oxygen from interlayer CO_3_^2−^ anions, respectively.^50,51^ The deconvoluted Bi 4f spectrum (Fig. 2(e)) shows peaks at 164.06 and 158.78 eV, assigned to Bi 4f_5/2_ and Bi 4f_7/2_, confirming the presence of Bi^3+^.^52,53^ In Fig. 2(f), the V 2p_3/2_ peak at 516.55 eV is attributed to V^5+^ species, while the peak at 531.68 eV corresponds to vanadium-oxygen bonding.^54^ Additionally, the peak observed at 284.83 eV in the C 1s spectrum (Fig. 2(g)) is assigned to C–C bonds originating from aliphatic groups, whereas the peaks at 286.34 and 288.83 eV are characteristic of C–O and C=O species, indicating the presence of CO_3_^2−^ in the composite.^55^ The high-resolution N 1s XPS spectrum of the composite in Fig. 2(h) is dominated by three component peaks at 399.65, 405.55, and 407.62 eV ascribed to urea-derived-N, NO_2_^−^ and NO_3_^−^ ions, indicating the role of nitrate ions as charge-balancing species.^56^ Collectively, these results affirm the effective synthesis and structural composition of the 5-NAL/BV nanohybrid.

### 3.2 Optical analysis

**Fig. 3:**
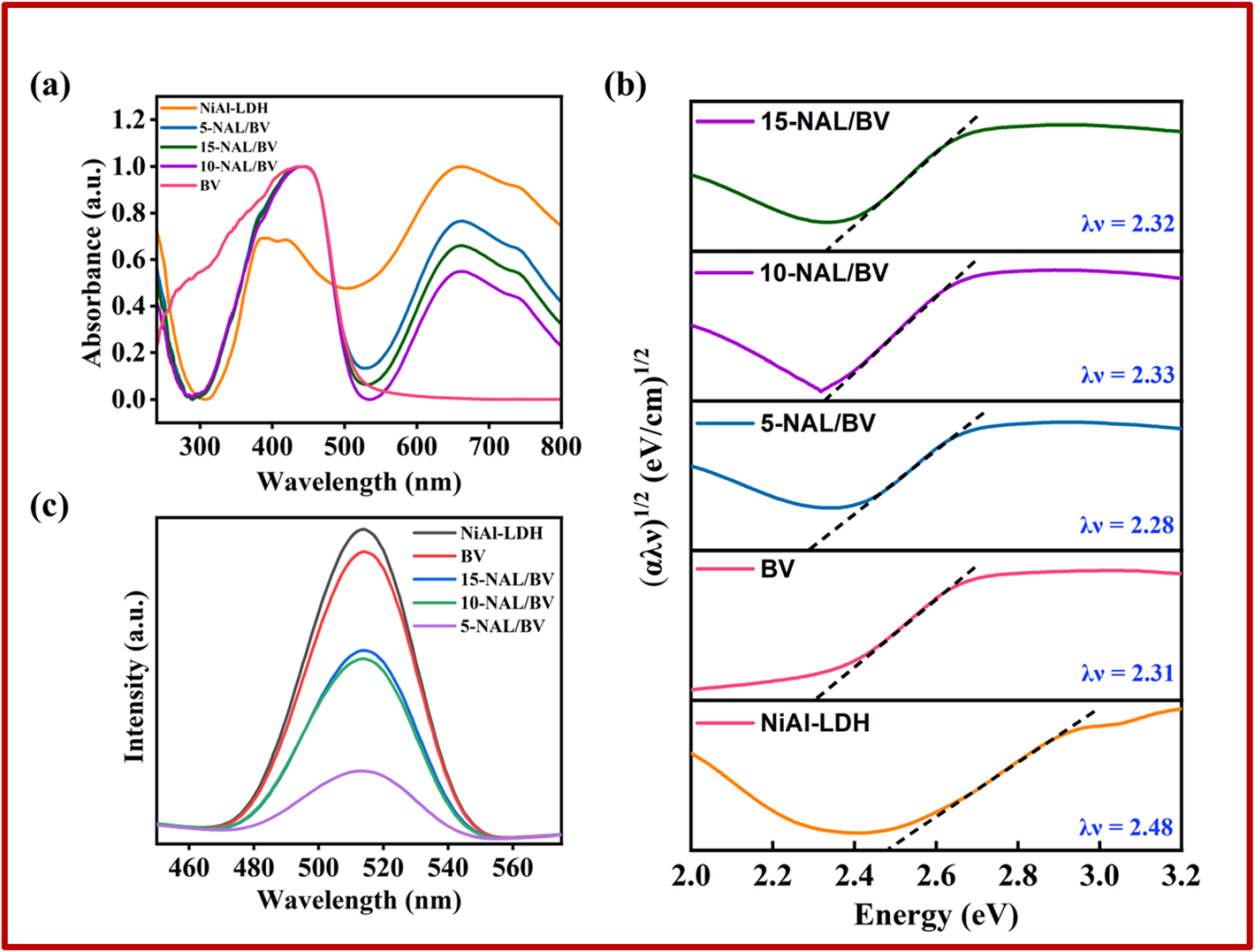
(a) UV-Vis DRS spectrum, (b) Tauc plot, and (c) PL spectra of the as-prepared photocatalysts.

Ultraviolet-visible diffuse reflectance spectroscopy (UV-vis DRS) was employed to assess the optical responsiveness of the synthesized photocatalysts, given that their photocatalytic efficiency is intrinsically linked to their light absorption characteristics. Fig. 3(a) displays the UV-visible absorption spectra of the developed photocatalysts. The optical band gap energies for NiAl-LDH, BV, and *x*-NAL/BV were estimated using Tauc plot approach, as outlined in eqn (3).

3 (*αλν*)^1/2^ = *A*(*λν* − *E*_g_)

where *α* denotes the absorption coefficient, *λ* represents Planck's constant, *ν* is the frequency of incident light, *A* refers to a proportionality constant, and *E*_g_ corresponds to the band gap energy determined from the intercept of the extrapolated linear region of the plot.^57^ The UV-visible absorption spectrum of BV reveals a broad absorption region extending over 350–450 nm, while NiAl-LDH exhibits three distinct absorption bands across the UV and visible regions: 200–300 nm, 300–500 nm, and 600–800 nm. The band in the UV range (200–300 nm) is attributed to ligand-to-metal charge transfer (LMCT) from the O 2p to the Ni 3d t_2g_ orbital. The bands in the visible region (300–800 nm) arise from d–d transitions typical of Ni^2+^ ions in an octahedral coordination environment. Specifically, the bands at 420 nm and 645 nm are attributed to spin-forbidden transitions ^3^A_2g_(F) → ^1^T_2g_(D) and ^3^A_2g_(F) → ^1^E_g_(D), while those at 380 nm and 740 nm correspond to spin-allowed transitions ^3^A_2g_(F) → ^3^T_1g_(P) and ^3^A_2g_(F) →^3^T_1g_(F), respectively.^58^ The integration of BiVO_4_ with NiAl-LDH induces a bathochromic shift, indicating a potential modification in the composite's electronic band structure. As illustrated in Fig. 3(b), the band gap energies of NiAl-LDH, BV, 5-NAL/BV, 10-NAL/BV, and 15-NAL/BV were determined to be 2.48, 2.31, 2.28, 2.33, and 2.32, in the same order. Notably, the 5-NAL/BV composite exhibited the lowest band gap, which is conducive to the formation of a higher density of photoexcited charge carriers, thereby enhancing its photocatalytic efficiency. Moreover, the reduced band gap of 5-NAL/BV renders it a promising candidate for effective solar light harvesting applications.

Photoluminescence (PL) emission serves as an effective method for evaluating the separation efficiency of photogenerated charge carriers in photocatalytic materials.^59^ Elevated PL intensity reflects increased electron–hole recombination, which reduces photocatalytic efficiency, while reduced PL intensity signifies enhanced charge separation and improved photocatalytic activity due to greater involvement of carriers in redox reactions.^60^ To analyze the PL spectra, a 400 nm excitation wavelength was employed, exhibiting a distinct emission peak at 514 nm for all photocatalysts, as illustrated in Fig. 3(c). The 5-NAL/BV sample demonstrates superior charge separation efficiency, as evidenced by its significantly lower PL intensity relative to other samples, including bare LDH, which exhibits the highest intensity. This enhanced charge separation directly correlates with a notable improvement in its photocatalytic performance. The electropositive surface of NiAl-LDH likely facilitates the retention of the photo-induced holes, thereby extending the lifetime of the charge carriers. Additionally, the disparity in Fermi energy levels between NiAl-LDH and BV likely contributes to a reduction in the recombination rates of photogenerated electrons and holes.^61^

### 3.3. Morphological analysis

**Fig. 4:**
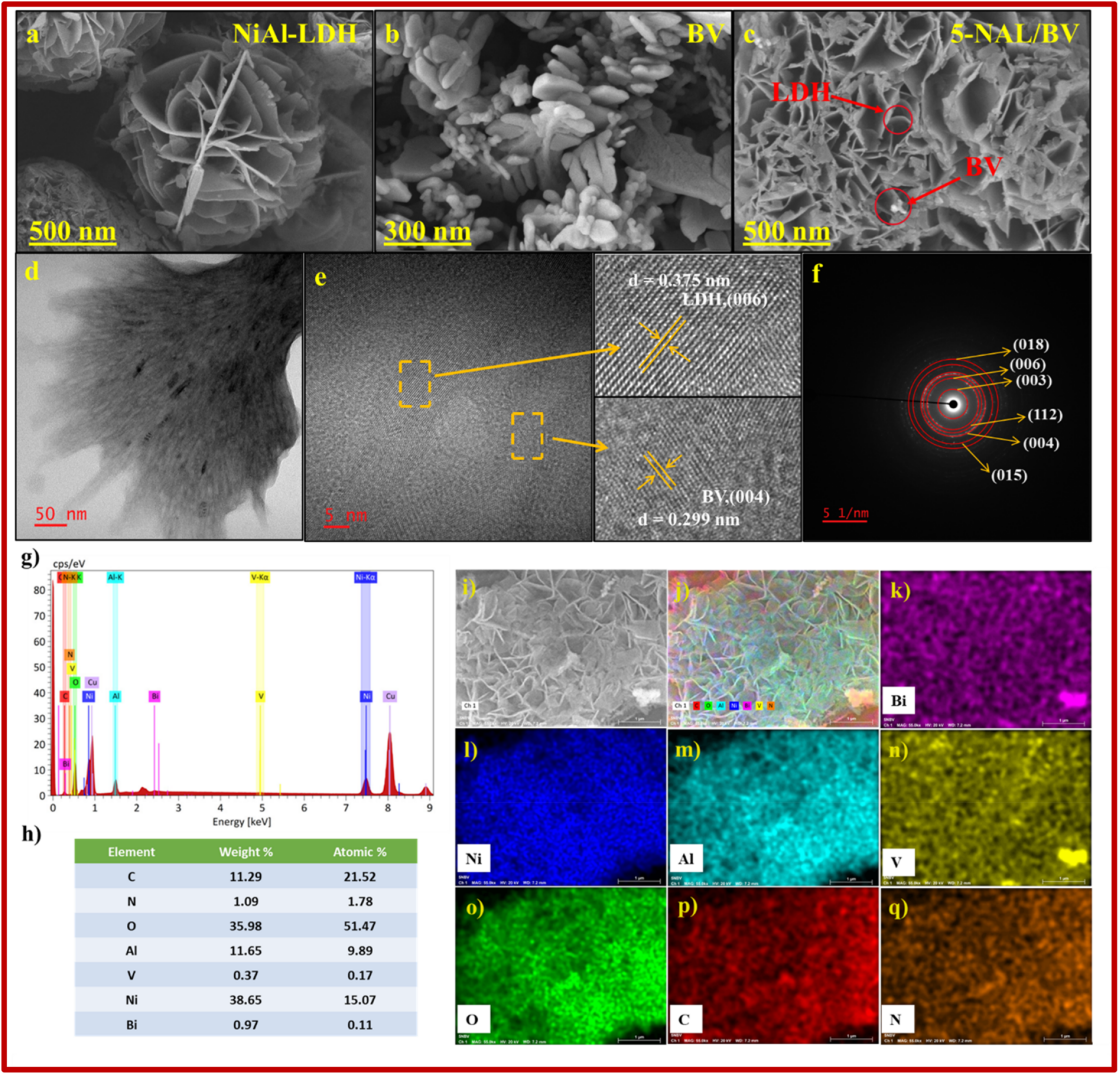
(a–c) FESEM images of bare LDH, bare BiVO_4_ and 5-NAL/BV nanocomposite, (d and e) HRTEM image, and (f) SAED pattern, (g–j) EDS spectrum of 5-NAL/BV composite, and (k–q) elemental mapping of major constituents, depicting their spatial distribution within the sample.

The surface morphologies of pure NiAl-LDH, BV, and 5-NAL/BV nanocomposite were characterized using FESEM (Field Emission Scanning Electron Microscope) analysis. The FESEM image of bare NiAl-LDH illustrates a three-dimensional flower-like structure consisting of a dense arrangement of ultrathin nanoflakes, while BV possesses an irregular disc-like shape (Fig. 4(a–c)).^62^ In the case of the 5-NAL/BV nanocomposite, 3D flower-like structures were observed alongside irregular disc-like shapes, thereby validating the effective fabrication of the 5-NAL/BV nanocomposite.

Energy-dispersive X-ray spectroscopy (EDS) was employed to determine the elemental constitution and assess the relative abundance of each constituent element within the sample.^63^ Elemental mapping images (Fig. 4(g–q)) confirm the homogeneous distribution of Ni, Bi, Al, V, C, O, and N elements throughout the sample matrix. Moreover, the lack of unexpected peaks in the EDS spectrum reinforces the compositional purity of the 5-NAL/BV surface, suggesting that no foreign elements were incorporated during the synthesis process.

High-resolution transmission electron microscopy (HRTEM) was conducted to analyze the microstructural characteristics of the fabricated material.^64^ A detailed analysis of the lattice fringes observed in the HRTEM images unveiled two distinct interplanar spacings of 0.375 nm and 0.299 nm. These values correspond to the (006) crystallographic planes of NiAl-LDH and the (004) planes of BiVO_4_, respectively, as shown in Fig. 4(d and e). These findings confirm the co-existence of NiAl-LDH and BiVO_4_ crystalline phases within the fabricated material. In Fig. 4(f), the Selected Area Electron Diffraction (SAED) pattern of 5-NAL/BV composite exhibits diffraction rings that indexed to the (003), (006), and (018) crystallographic planes of NiAl-LDH whereas the (112), (004), and (015) planes correspond to those of BiVO_4_, which affirms the effective integration of LDH and BV in the fabricated composite.

### 3.4 Surface area analysis

Nitrogen sorption isotherms were employed to assess specific surface area (*a*_BET_) and porosity of NiAl-LDH, BV, and *x*-NAL/BV nanocomposites, as illustrated in Fig. S1(a), while Barrett–Joyner–Halenda (BJH) method was utilized to estimate the mean pore size distribution, as shown in Fig. S1(b). The adsorption isotherms obtained for the photocatalysts were classified as type IV, exhibiting a distinct H3 type hysteresis loop at higher relative pressures (*P*/*P*_o_). This behavior is typically associated with mesoporous frameworks, implying the presence of well-organized and uniformly distributed pore structures within the material.^65–67^ The *a*_BET_, total pore volume, and mean pore diameter of the samples are provided in Table 1. Among the fabricated photocatalysts, the 5-NAL/BV nanocomposite exhibited higher surface area (38 m^2^ g^−1^), which may be ascribed to the structural contributions of the heterojunction interfaces. This increased surface area provides abundant active sites, thereby facilitating improved photocatalytic performance.

**Table 1:** Comparison of specific surface area, total pore volume, and mean pore diameter of fabricated catalysts

Sample	Specific surface area (m^2^ g^−1^)	Total pore volume (cm^3^ g^−1^)	Mean pore diameter (nm)
NiAl-LDH	39.5	0.149	15.6
BV	5.46	0.0126	9.2
5-NAL/BV	38	0.1472	7.3
10-NAL/BV	36.2	0.1419	15.7
15-NAL/BV	28.5	0.0517	15.5

### 3.5 Electrochemical impedance analysis

EIS measurements were conducted in the dark for NiAl-LDH, BV, and 5-NAL/BV to investigate charge separation. The Nyquist plots (Fig. S2) reveal that 5-NAL/BV exhibits a much smaller semicircle radius compared to NiAl-LDH and BV, indicating lower charge transfer resistance and more efficient electron transport. Fitting the data to the equivalent circuit using EC-Lab software yielded the parameters summarized in Table S1. Notably, the 5-NAL/BV sample shows a lower charge transfer resistance (1.66 Ω), confirming that incorporating BV into NiAl-LDH enhances interfacial charge dynamics, thereby improving the photocatalytic performance of the composite.

### 3.6 Photocatalytic study

#### 3.6.1 Optimum catalyst dose

**Fig. 5:**
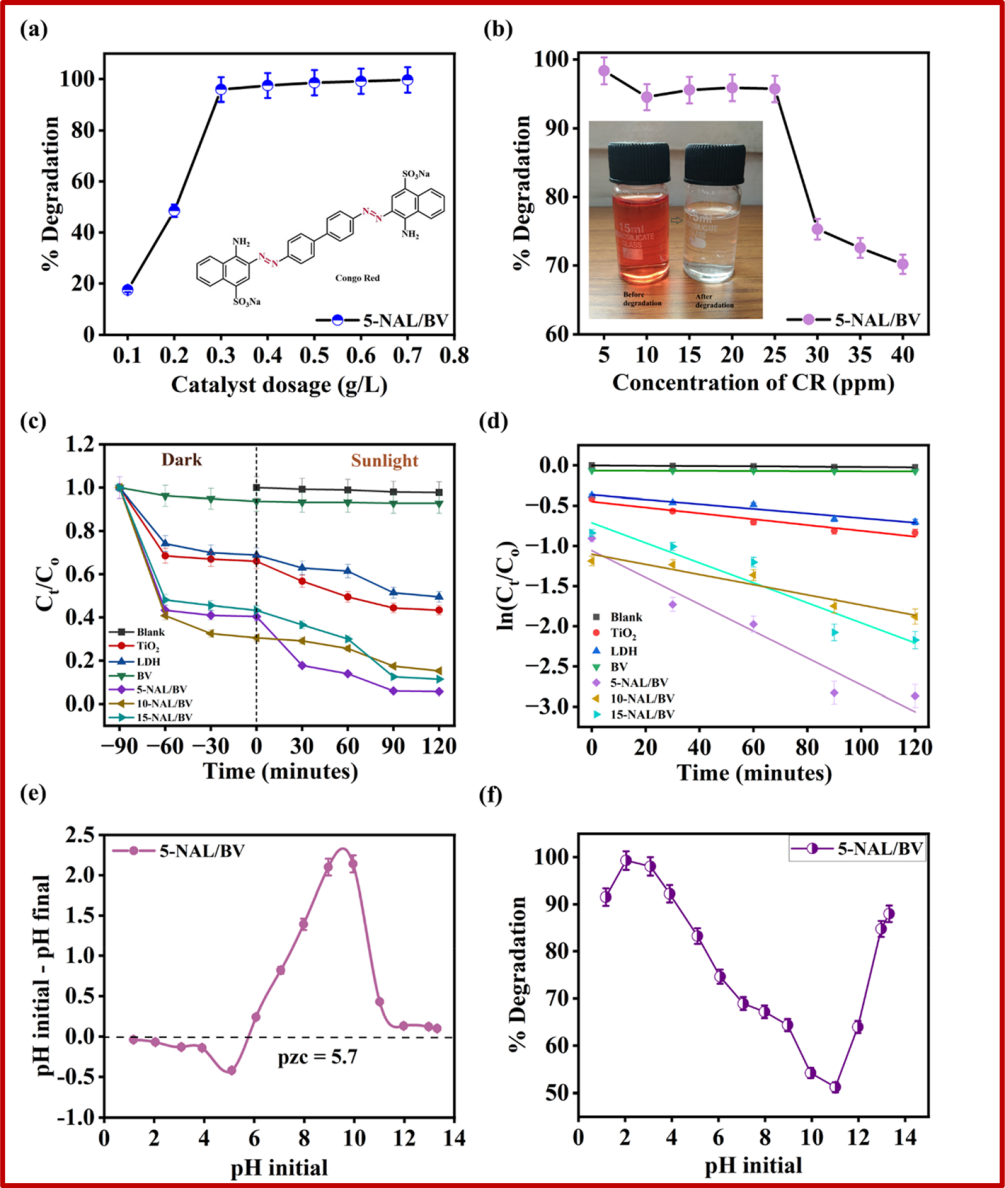
(a and b) Effect of various factors [amount of catalyst loading, and pollutant concentration] on degradation efficiency, (c) assessment of photocatalytic activity of the prepared photocatalysts for CR decomposition, (d) corresponding pseudo-first-order kinetic plots, (e) pzc of the 5-NAL/BV photocatalyst, and (f) variation in photocatalytic behavior with pH.

Optimizing catalyst loading is crucial in photocatalysis to balance degradation efficiency with material economy.^68^ For this, the photocatalytic degradation of CR was investigated as a function of catalyst dose by adding varying amounts of 5-NAL/BV to 10 mL of CR solution (25 ppm) under visible light irradiation with continuous stirring for 2 hours. As seen in Fig. 5(a), a gradual increase in catalyst dosage from 0.1 g L^−1^ to 0.7 g L^−1^ led to a significant improvement in degradation efficiency. Notably, raising the photocatalyst dose from 0.1 to 0.3 g L^−1^ significantly enhanced CR deterioration from 17.49% to 95.9%, attributed to an increase in active surface sites and reactive oxygen species (OH and O_2_·^−^) generation.^69^ However, beyond this concentration (0.3 to 0.7 g L^−1^), the efficiency reached a saturation point. This phenomenon may be ascribed to enhanced opacity and scattering resulting from the higher catalyst concentration in the solution.^70^ Considering the reduction efficiency, a catalyst dose of 0.3 g L^−1^ was identified as the optimal level for subsequent investigations. This finding suggests that a relatively small amount of photocatalyst is adequate to facilitate the photocatalytic breakdown of hazardous pollutants.

#### 3.6.2 Pollutant concentration

In general, photocatalytic activity exhibits an inverse relationship with initial pollutant concentration due to competitive adsorption at limited catalytic active sites. At elevated concentrations, the dye dominates photon absorption, reducing light availability for catalyst activation and impairing degradation kinetics.^71^ This effect was investigated systematically by varying CR concentrations from 5 to 40 mg L^−1^, while maintaining all other experimental parameters constant. As represented in Fig. 5(b), degradation efficacy declined significantly beyond 25 ppm, suggesting a concentration threshold for optimal photocatalytic activity. This decline may be associated with the accumulation of reactive intermediates generated during dye breakdown, which compete with parent molecules for active sites.^72^

#### 3.6.3 Kinetics

To evaluate the effectiveness of the prepared photocatalysts, a light-induced degradation experiment was carried out by exposing a 10 mL, 25 ppm CR solution to sunlight for 120 minutes without a catalyst, resulting in only a 2.2% degradation of the dye. Photocatalytic degradation results (Fig. 5(c) and S3(a–f)) showed that bare NiAl-LDH and BV decomposed up to 50.5% and 7.1%, respectively, whereas 5-NAL/BV exhibited a remarkable 94.3% efficiency, potentially ascribed to improved interfacial charge migration within the photocatalyst. Conversely, the degradation efficiency of commercial TiO_2_-P25 was tested under identical conditions, achieving a maximum of 56.6%, which remained inferior to that of the prepared photocatalysts. These findings validate that the incorporation of 5 wt% BV into the LDH matrix effectively boosted the photocatalytic efficiency of the resulting material. The reaction rate of photocatalytic degradation data was further evaluated using pseudo-first-order kinetics, which is expressed by eqn (4).4
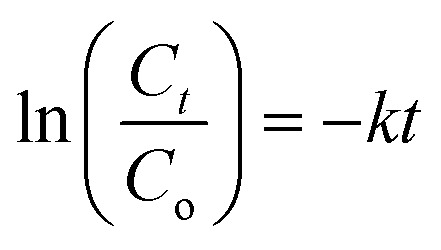
In this expression, *k* represents the degradation rate constant determined by applying a linear fit to the data, *t* denotes the time (minutes), *C*_o_ indicates the initial pollutant concentration, whereas *C*_*t*_ signifies the residual concentration at different time intervals during catalytic degradation.^73^ As illustrated in Fig. 5(d), among the synthesized hybrids, 5-NAL/BV exhibited the highest rate constant (0.01673 min^−1^), significantly outperforming 15-NAL/BV (0.01247 min^−1^) and 10-NAL/BV (0.00634 min^−1^). Also, when compared to pure LDH (0.00287 min^−1^), BV (0.00008 min^−1^), and commercial TiO_2_-P25 (0.00362 min^−1^), the hybrid exhibited remarkable efficiency, reinforcing the impact of structural modifications. The hybridisation of NiAl-LDH with BV generates a synergistic effect, which can be quantified by the synergy factor (*R*) as expressed in eqn (5).5
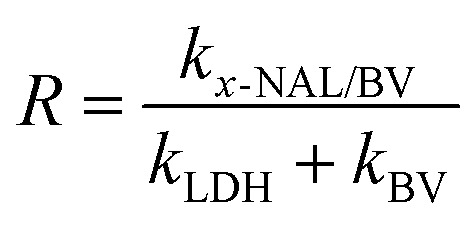
Here, *k*_*x*-NAL/BV_, *k*_LDH,_ and *k*_BV_ correspond to the rate constants of the prepared hybrids, bare LDH, and bare BV, respectively. With the highest *R* factor (5.67), 5-NAL/BV exhibited the superior efficiency due to its strong synergistic effect on LDH and BV. Table 2 provides a comparative evaluation of the photocatalysts' degradation efficiency, rate constants, and synergy factor.

**Table 2:** Comparison of degradation efficacy, rate constant, and synergy factor of various synthesized photocatalysts

Photocatalysts	(%) Degradation	Kinetic rate constant (min^−1^)	Synergy factor (R)
TiO_2_-P25	56.66	0.00362	—
NiAl-LDH	50.5	0.00287	—
BV	7.1	0.00008	—
5-NAL/BV	94.3	0.01673	5.67
10-NAL/BV	84.8	0.00634	2.15
15-NAL/BV	88.7	0.01247	4.23
No catalyst	2.2	0.00019	—

#### 3.6.4 Effect of pH

The photocatalyst's adsorption behavior is strongly influenced by the pH of the solution, which plays a crucial role in modulating its photocatalytic degradation efficiency. To assess this effect, the pH of the CR solution was adjusted by using 0.5 N NaOH and 0.5 N HCl solutions and tested under controlled photocatalytic conditions. In Fig. 5(f), a marked decline in the photocatalytic degradation efficiency of CR, from 99.27% to 51.19%, is observed as the solution pH increases from 2 to 11. The pH-responsive surface properties of the as-prepared 5-NAL/BV photocatalyst significantly influence its interaction with dye molecules. This behavior is governed by the point of zero charge (pzc), which for 5-NAL/BV is approximately 5.7 (Fig. 5(e)). At pH < pzc, the catalyst surface becomes protonated, strengthening electrostatic affinity for negatively charged CR molecules and enhancing photocatalytic degradation. In contrast, at pH > pzc, the surface acquires a negative charge, leading to repulsion and reduced degradation efficiency. Moreover, acidic conditions favour the formation of reactive species such as hydroxyl radicals under light irradiation, whereas alkaline media tend to scavenge these species, further diminishing activity.^74,75^ Although maximum photocatalytic efficiency is typically achieved at low pH values (around pH 2), the experiments in this study were conducted within a pH range of 5–6 to better mimic environmentally realistic conditions.

#### 3.6.5 Influence of radiative sources

**Fig. 6:**
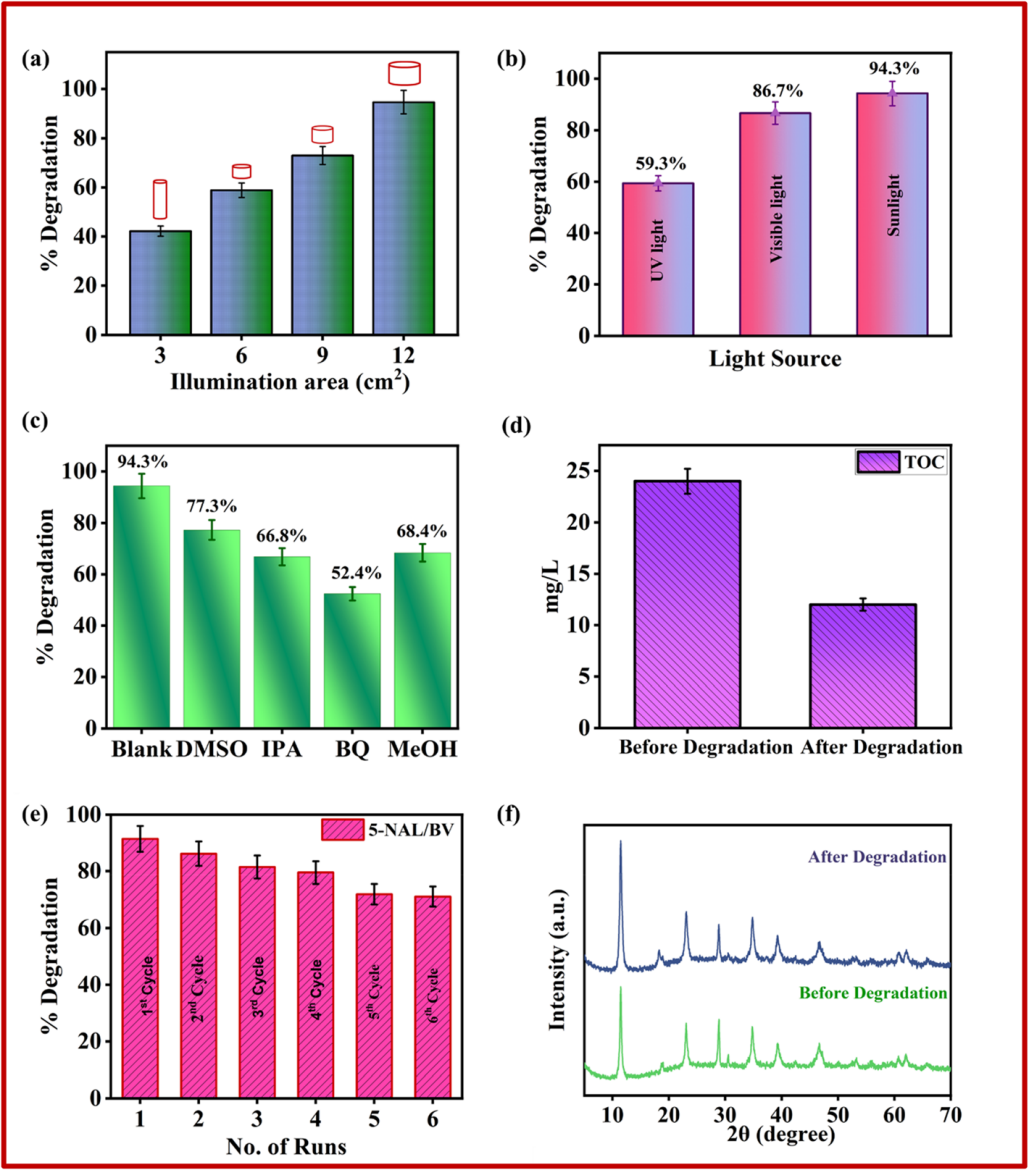
(a–c) Impact of illuminating area, source of light, and different scavenging agents on CR degradation, (d) total organic carbon analysis, (e) reusability test of 5-NAL/BV catalyst over six consecutive cycles, and (f) XRD pattern of used catalyst.

Under optimized experimental conditions, a comparative study was carried out employing ultraviolet, visible, and natural sunlight irradiation to evaluate their influence on photocatalytic activity. As shown in Fig. 6(a), CR degradation reached 59.3% in UV light and 86.7% in visible light. Interestingly, the peak degradation efficiency of 94.3% was attained when employing natural sunlight in conjunction with the 5-NAL/BV photocatalyst. These outcomes highlight the potential of natural sunlight in driving dye decomposition through the synthesized photocatalysts.

#### 3.6.6 Illumination area

The impact of the illuminated surface area on the photocatalytic efficiency was assessed by employing reaction vessels with varying cross-sectional area to regulate the exposed reactive interface, while maintaining all other experimental conditions constant. Fig. 6(b) clearly illustrates a positive correlation between the exposed surface area and degradation efficiency, indicating that increased light exposure enhances photocatalytic performance. This is because a larger illuminated area allows greater light absorption by the reaction mixture, thereby promoting more efficient degradation of pollutants.^76^

#### 3.6.7 Scavenging study

A scavenger study was conducted using typical quenching agents to investigate the involvement of reactive species in the photocatalytic mechanism. For this, different scavengers like dimethyl sulfoxide (DMSO) and isopropyl alcohol (IPA) were used to scavenge hydroxyl radicals (˙OH), benzoquinone (BQ) for superoxide anions (O_2_˙^−^), and methanol (MeOH) for trapping holes (h^+^). During the study, 1 mM solutions of the respective scavengers were prepared in 25 ppm CR, and the tests were carried out under the same conditions as the photocatalytic experiments. Fig. 6(c) clearly indicates that the highest degradation efficiency of CR, recorded at 94.3%, was attained without the introduction of any scavenging agents. However, upon the addition of BQ, the photocatalytic efficiency of the 5-NAL/BV composite was notably suppressed, with the degradation efficiency decreasing significantly to 52.4%. This observation highlights the critical role of O_2_˙^−^ as the active radical species contributing to the breakdown of CR. In contrast, methanol and IPA exhibited comparable but less significant effects (67%) on the degradation process, indicating that both h^+^ and ˙OH contribute minimally yet crucially in overall dye removal.^77,78^

#### 3.6.8 Total organic carbon

The evaluation of organic matter mineralization, in conjunction with the degradation of organic contaminants, is of significant importance. To quantify the mineralization of CR, an analysis of total organic carbon was conducted. The elevated levels of TOC implied that the CR dye comprised a considerable amount of organic matter. After 120 minutes of photocatalytic exposure to sunlight in the presence of the as-prepared photocatalyst, a 50% reduction in TOC was achieved, indicating significant degradation and mineralization of organic compounds (Fig. 6(d)). At low pollutant concentrations or in the absence of stable intermediates, degradation and mineralization exhibit comparable half-lives. However, at elevated concentrations where persistent intermediates form, mineralization proceeds at a slower rate than the degradation of the parent compound.^79^ Thus, it can be inferred that half of the initial organic carbon was mineralised, while the remaining fraction persists in the form of intermediates possessing limited potential for further mineralization.^80^

#### 3.6.9 Structural integrity and reusability

**Fig. 7:**
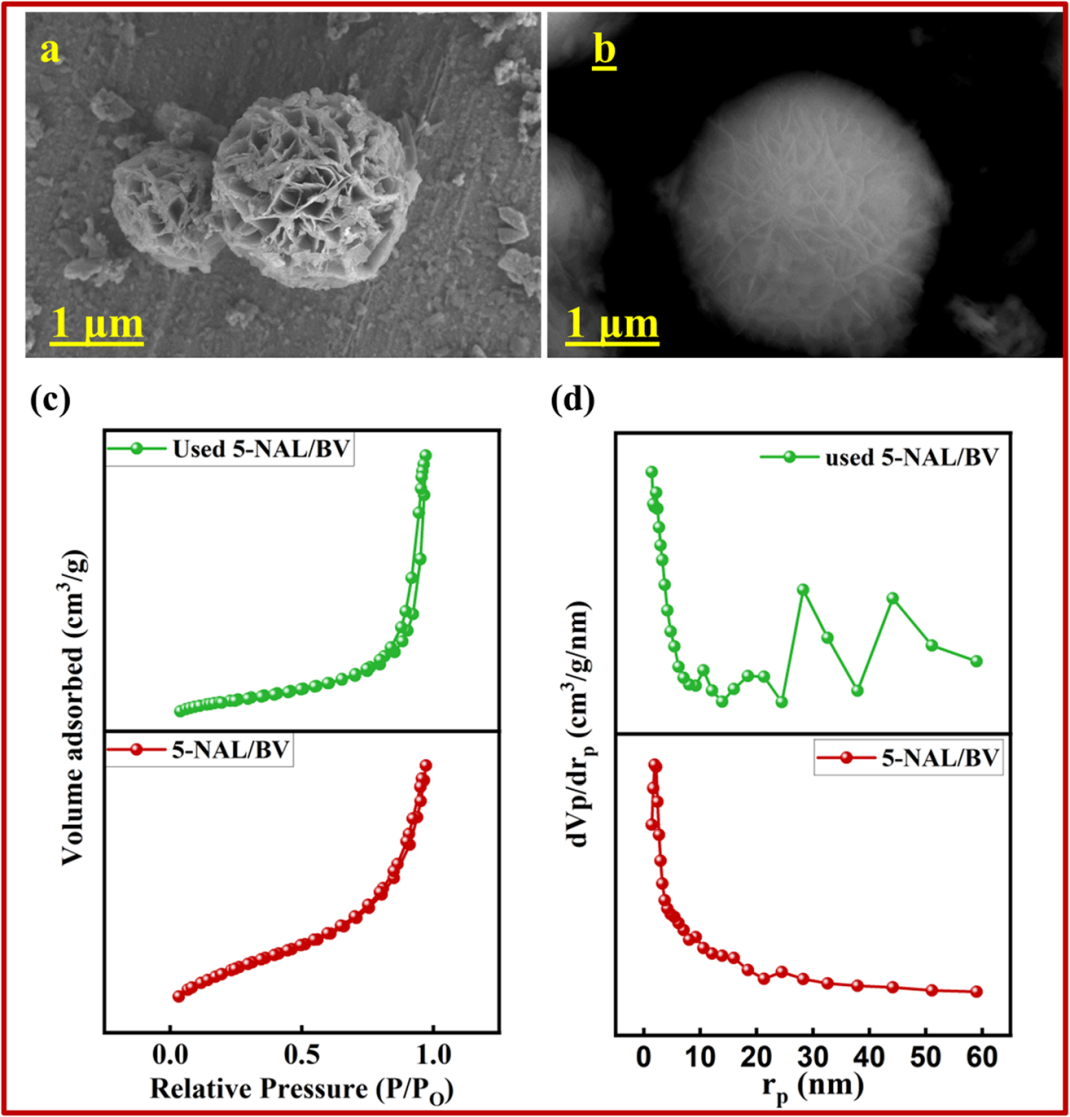
(a) FESEM and (b) SEM image of the used 5-NAL/BV nanocomposite, comparison of (c) nitrogen sorption isotherms, and (d) BJH pore size distribution plots of the fabricated photocatalyst before and after degradation.

Sustained reusability and optical resilience of photocatalysts are essential criteria for their practical implementation in environmental remediation. In this context, the recyclability and operational stability of the 5-NAL/BV photocatalyst were evaluated through successive recycling experiments over six cycles during the degradation of CR. Before each subsequent cycle, the photocatalyst was centrifuged and dried at 50 °C for 24 hours. The results (Fig. 6(e)) indicated a decrease of around 20% in the photocatalytic efficiency of the 5-NAL/BV nanohybrid. This reduction may be due to the loss of material during the washing process, which could have reduced its surface catalytic properties. Also, the aggregation of nanocomposites and subsequent occlusion of active sites by CR and its intermediates leads to a gradual decline in the surface area and adsorptive capacity of the photocatalysts, resulting in attenuated photocatalytic activity. Nevertheless, the reduction in photocatalytic activity remained minimal even after six extended runs, confirming that the 5-NAL/BV composite is both reusable and highly effective for photocatalytic applications.^81,82^ Furthermore, XRD, FTIR, BET, and SEM analyses were carried out to assess the structural robustness and morphology of the recycled nanocomposite after extended light exposure. As depicted in Fig. 6(f), the XRD peaks of both fresh and recycled nanocomposite exhibits no significant structural changes. Similarly, FESEM and SEM images (Fig. 7(a and b)) and FTIR curves (Fig. S4) reveal no noticeable alterations in morphology and structural integrity. The minimal change in specific surface area (Fig. 7(c)) and pore size distribution (Fig. 7(d)) after degradation indicates that the photocatalyst retains its original textural characteristics, thereby confirming the nanocomposite's stability and reusability for practical applications.

#### 3.6.10 Comparative efficiency analysis against literature reports

A comprehensive literature review has been conducted to compare the efficiency of various photocatalysts in removing CR, as summarized in Table 3. It is worth emphasizing that, except for a few reported catalysts, the present system exhibits equivalent or superior photocatalytic efficiency. However, the majority of these comparative systems necessitate either elevated catalyst dosages^83,84^ or reduced pollutant concentrations^85–88^ to attain comparable degradation performance. Such comparisons indicate that employing the 5-NAL/BV nanohybrid as a photocatalyst offers an effective and practical strategy for the catalytic removal of azo dyes.

**Table 3:** A critical comparison of photocatalytic efficiencies for CR decomposition between NiAl-LDH/BiVO_4_ hybrid catalyst and other photocatalysts reported in the literature

Sr. No.	Photocatalysts	Catalyst loading (mg L^−1^)	Pollutant initial conc. (ppm)	Duration (min)	Light source	Degradation (%)	Ref.
1	TiO_2_	400	4 ppm	30 min	Sunlight	64.72%	83
2	*m*-BiVO_4_/*t*-BiVO_4_	150	5 ppm	60 min	300 W Xenon lamp	98%	85
3	MXene/BiVO_4_/Bi_2_S_3_	430	7 ppm	70 min	400 W tungsten lamp	92.5%	89
4	ZIF-8@BiVO_4_	600	20 ppm	90 min	Sunlight	94.4%	84
5	NiAl-SiW_12_O_40_	100	20 ppm	120 min	UV light	86%	90
6	Co/Na BiVO_4_	200	5 ppm	60 min	Sunlight	85%	86
7	NiAl/AC	100	10 ppm	40 min	UV light	91.2%	87
8	BiVO_4_/MXene	100	10 ppm	60 min	300 W Xenon lamp	99.5%	88
9	PANI/BiOCl	100	10 ppm	60 min	225 W Xe/400 nm filter	88.35%	91
10	NiAl-LDH/BiVO_4_	300	25 ppm	120 min	Sunlight	94.3%	This work

#### 3.6.11 Photocatalytic mechanism

**Scheme 2:**
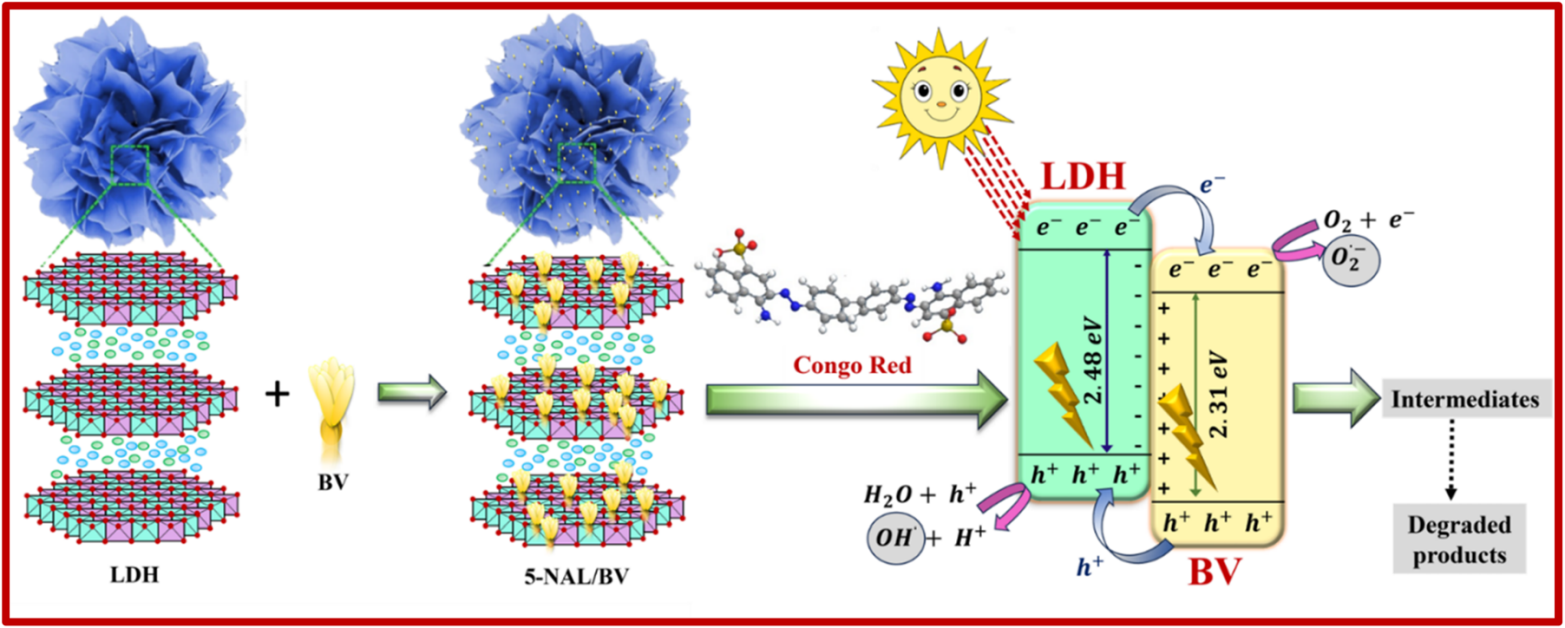
Illustrative overview of the proposed mechanism for CR degradation *via* photocatalysis over the 5-NAL/BV composite.

The efficiency of a semiconductor in photocatalytic processes is significantly governed by the energies of its valence and conduction bands. Using Mulliken's electronegativity theory, the conduction band potential at the point of zero charge can be theoretically estimated by eqn (6).6
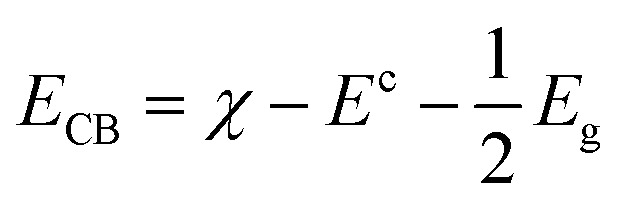
In this formulation, *E*_CB_ refers to the conduction band edge potential, *E*_g_ indicates the semiconductor's band gap energy, and *χ* signifies its absolute electronegativity. The term *E*^c^ corresponds to the energy of a free electron relative to the hydrogen electrode, typically considered to be around 4.5 eV. The potential at the edge of valence band (*E*_VB_) can be estimated using the relation *E*_VB_ = *E*_g_ + *E*_CB_. According to literature reports, BiVO_4_ possesses an absolute electronegativity of 6.04 eV.^92^ Using this parameter, the corresponding band edge potentials are derived, with the conduction band positioned at approximately 0.385 eV and the valence band at around 2.695 eV. No doubt, this approach is theoretically significant; however, it is rarely applied to NiAl-LDH, owing to the substantial difficulties in precisely determining its absolute electronegativity, which stem from its complex layered structure. Therefore, the conduction band potential of NiAl-LDH was estimated by referencing the valence band potential value of 1.70 eV, as reported in the literature.^93^ Utilizing this value in conjunction with the optical band gap of NiAl-LDH (2.48 eV), as determined from Tauc plot analysis, the conduction band potential was estimated to be approximately −0.78 eV. The band alignment between NiAl-LDH and BV satisfies the energetic criteria necessary for the formation of a Z-scheme heterojunction. This conclusion is further corroborated by the outcomes of radical trapping experiments, photoluminescence, and EIS analysis of 5-NAL/BV composite, which indicate dominant radical formation, suppressed recombination rate, and lower charge transfer resistance. Together, these findings provide compelling evidence for the operation of a direct Z-scheme rather than a conventional type-II mechanism. Based on these findings, a plausible Z-charge transfer mechanism accounting for the superior performance of 5-NAL/BV nanocomposite has been formulated, which is schematically presented in Scheme 2.

Upon sunlight irradiation, both NiAl-LDH and BV generate photocarriers, producing electrons in the conduction band (CB) and holes in the valence band (VB). The direction of charge transfer is dictated by the relative positions of their band edge potentials. Due to the more negative potential of its CB (−0.78 eV), LDH readily donates photogenerated electrons to the CB of BV, which lies at 0.385 eV. Concurrently, the higher valence band potential of BV (2.69 eV) relative to LDH (1.70 eV) offers a suitable energetic gradient that promotes the transfer of holes from BV to LDH. The effective separation of photoinduced carriers is further supported by TRPL (Time-Resolved Photoluminescence) analysis (Fig. S5 and Table S2), where 5-NAL/BV exhibits a longer average lifetime (0.223 ns) than NiAl-LDH (0.154 ns) and BiVO_4_ (0.205 ns). This prolonged lifetime confirms that the interfacial heterojunction efficiently suppresses e^−^–h^+^ recombination, providing experimental evidence for the proposed Z-scheme charge transfer pathway. At the photocatalyst's surface, photogenerated electrons reduce molecular oxygen (O_2_) to yield O_2_˙^−^, while the accumulated h^+^ oxidize water molecules to produce ˙OH and protons (H^+^). These reactive species (ROS) actively participate in the oxidative degradation of CR, leading to stepwise breakdown into intermediates and ultimately mineralized products (CO_2_ and H_2_O), as shown in the following reactions.^94^NiAl LDH + *λν* → NiAl LDH(h_VB_^+^ + e_CB_^−^)BV + *λν* → BV(h_VB_^+^ + e_CB_^−^)NiAl LDH (e_CB_^−^) → BV(e_CB_^−^) + O_2_ → O_2_˙^−^BV(h_VB_^+^) → NiAl LDH(h_VB_^+^) + H_2_O → OH^˙^ + H^+^
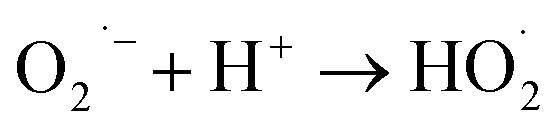

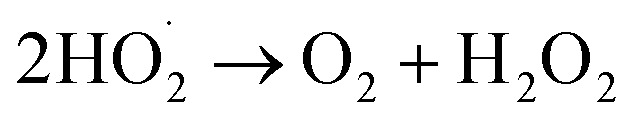
H_2_O_2_ + e_CB_^−^ → OH^˙^ + OH^−^O_2_˙^−^ + OH^˙^ + CR → intermediates → mineralized products

This Z-scheme pathway not only facilitates efficient redox reactions but also preserves the strong oxidation potential of BV and the strong reduction potential of NiAl-LDH, accounting for the superior degradation efficiency of the 5-NAL/BV heterojunction. Furthermore, the stability of this band alignment is consistent with the excellent recyclability of the photocatalyst, underscoring its promise for practical wastewater treatment.

#### 3.6.12 High-resolution mass spectrometry

**Scheme 3:**
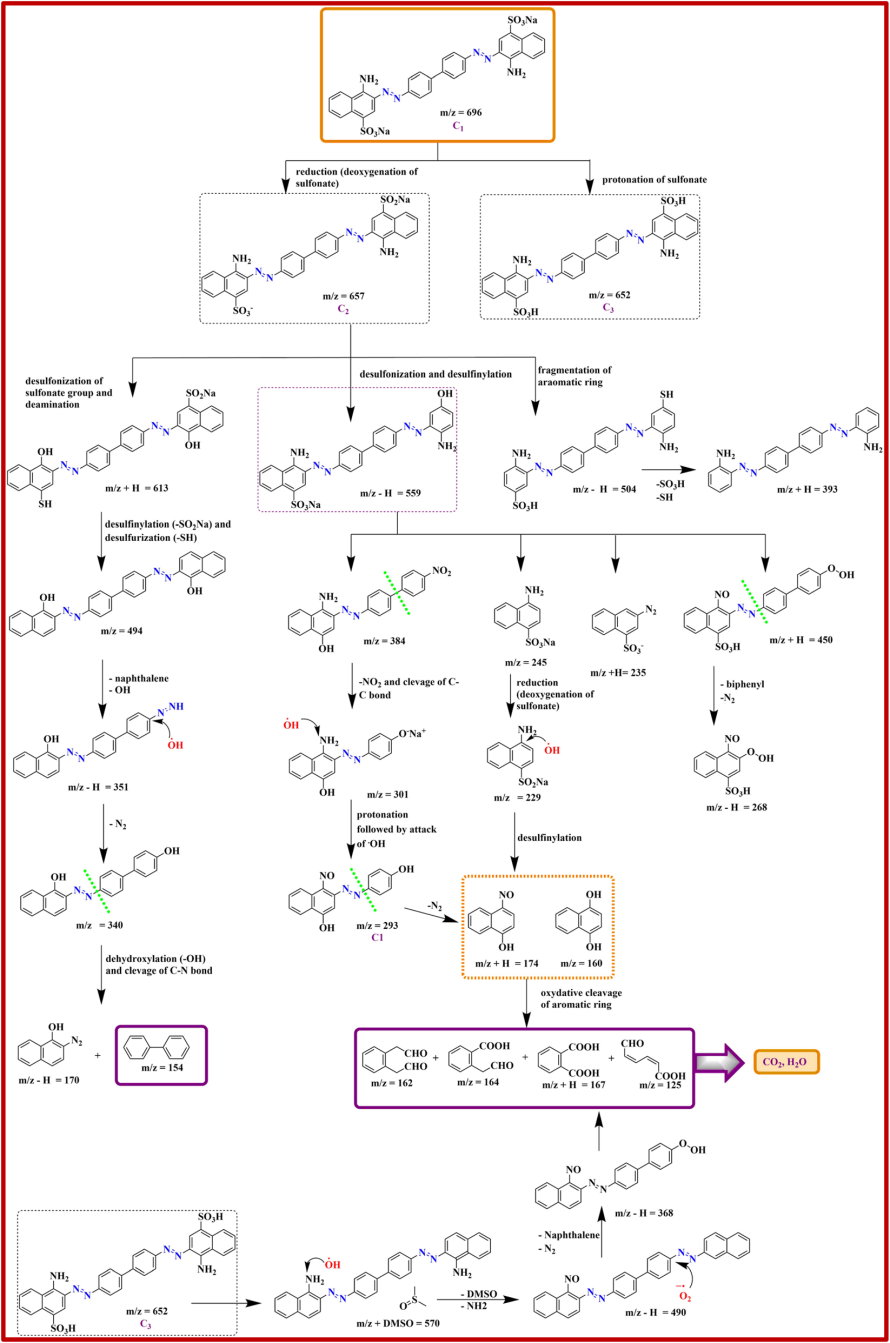
Potential reaction pathway governing the photocatalytic decomposition of CR under sunlight exposure in the presence of 5-NAL/BV composite.

A detailed investigation of the intermediates was conducted using HRMS, with the corresponding results shown in Scheme 3 and Fig. S6. The signal observed at *m*/*z* 696 is attributed to the molecular ion peak of the parent CR molecule (*C*_1_). The CR decomposition involves several processes, including desulfonization, de-amination, cleavage of C–N and C–C bonds, and fragmentation of the benzene ring. Firstly, the deoxygenation of one sulfonate group leads to the intermediate (*C*_2_) with *m*/*z* 657, while protonation of the parent molecule gives intermediate (*C*_3_) with *m*/*z* value of 652. The intermediate *C*_2_ gives rise to secondary fragments with *m*/*z* 613, 559, and 504 through desulphonation, desulfinylation, and fragmentation of the aromatic ring. Subsequent degradation of these fragments ultimately results in final products with *m*/*z* values of 125, 154, 160, 162, and 167. Likewise, the intermediate *C*_3_ upon degradation leads to low molecular mass fragments, which show peaks at 570, 490, and 368. Continued breakdown of these fragments obtained from photocatalysis of *C*_2_ and *C*_3_ intermediates leads to complete mineralization, yielding H_2_O and CO_2_, highlighting the effectiveness of 5-NAL/BV hybrid.

## 4. Conclusions

In this work, NAL/BV Z-scheme heterojunction nanocomposites with varying BV content have been successfully synthesized through an *ex situ* assembly approach and characterized by different spectroscopic techniques. These composites were used for solar-driven photocatalytic decomposition of Congo red. Amongst the synthesized nanocomposites, 5-NAL/BV manifested superior photocatalytic proficiency, exhibiting pseudo first-order kinetics with a rate constant of 0.01673 min^−1^, surpassing those of LDH, BV, and TiO_2_-P25, with corresponding factors of 5.8, 209, and 4.6 due to higher surface area and high synergy factor. The superior efficiency of this composite is primarily associated with the pronounced synergistic interaction between LDH and BV, which facilitates suppressed e^−^–h^+^ recombination, enhanced charge separation, and rapid migration of photoinduced charge carriers. Also, the fabricated heterojunctions demonstrated excellent photostability, consistently maintaining their catalytic effectiveness over multiple cycles with no evident loss in activity. This study offers a valuable reference framework for employing Z-scheme heterojunction photocatalysts in the remediation of dye-based organic contaminants. Future work could extend this strategy to diverse emerging organic pollutants under real wastewater conditions, while the application of advanced *in situ* characterization techniques would provide deeper insights into the charge transfer mechanisms. Ultimately, such efforts will accelerate the translation of laboratory-scale findings into sustainable photocatalytic technologies for real-world environmental remediation.

## Author contributions

Manpreet Kaur: conceptualization, methodology, data collection, data analysis and writing the original draft. Pritam Hait: data analysis and co-wrote the paper. Soumen Basu: supervision, data investigation, data validation, reviewing, and editing.

## Conflicts of interest

The authors declare that they have no known competing financial interests or personal relationships that could have appeared to influence the work reported in this paper.

## Supplementary Material

RA-015-D5RA06146F-s001

## Data Availability

The data supporting the findings of this study are accessible from the corresponding authors upon reasonable request. Supplementary information is available. See DOI: https://doi.org/10.1039/d5ra06146f.
