# Supplementary material for: Flower-like NiAl-LDH/BiVO4 Z-scheme photocatalysts for sunlight-driven degradation of azo dye: performance and mechanistic insights
Source: RSC Adv. 2025 Oct 7;15(44):37166–82. doi: 10.1039/d5ra06146f (PMC12501841; doi:10.1039/d5ra06146f)
Supplement: RA-015-D5RA06146F-s001 [file RA-015-D5RA06146F-s001.pdf]

Supplementary Information (SI) for RSC Advances.
This journal is © The Royal Society of Chemistry 2025

Supplementary Materials

Flower-like NiAl-LDH/$BiVO_4$ Z-Scheme Photocatalysts for Sunlight-Driven Degradation of Azo Dye: Performance and Mechanistic Insights

Manpreet Kaur[a], Pritam Hait[a,b], Soumen Basu[a,b*]

[a]Department of Chemistry and Biochemistry, Thapar Institute of Engineering and Technology (TIET), Patiala-147004, Punjab, India

[b]TIET-Virginia Tech Center of Excellence in Emerging Materials, Thapar Institute of Engineering and Technology (TIET), Patiala-147004, Punjab, India

*Corresponding author: E-mail: soumen.basu@thapar.edu (Prof. Soumen Basu)

S1. Materials

The following chemicals were used without further purification: nickel nitrate hexahydrate (98%), aluminium nitrate nonahydrate (98%), ammonium fluoride (95%), urea (99.5%), bismuth nitrate pentahydrate (98%), ammonium metavanadate (99%), sodium dodecyl benzene sulphonate (80%), and sodium hydroxide pellets (97%), all procured from Loba Chemie, India. Nitric acid (69%), double-distilled water, and absolute ethanol (99.9%) were used for solution preparation.

S2. Characterization methods

The X-ray diffraction (XRD) analysis of the materials was performed using a PAN Analytical X` Pert-Pro X-ray diffractometer, operated at 45 kV with Cu Kα radiation. The scan range was 5-90° with a step size of 0.02°, and the wavelength was 1.5406 Å. UV–Vis diffuse reflectance spectroscopy (DRS) was performed by using a JASCO V-750 spectrophotometer in diffuse absorbance mode to investigate the optical properties of the synthesized photocatalysts. In contrast, a Shimadzu UV-2600 was employed to monitor the photocatalytic degradation kinetics. Photoluminescence (PL) measurements were obtained using a Shimadzu RF-6000 spectrofluorophotometer with an excitation wavelength of 400 nm. The electronic states of the elements and their surface composition in the synthesized samples were analysed using a ThermoFisher (NEXSA) X-ray photoelectron spectrometer equipped with an Al Kα X-ray source. Nitrogen sorption measurements were conducted utilizing a Microtrac Belsorp Mini-II surface area analyzer (Bel Japan, Inc.). Before analysis, the samples were subjected to pre-treatment under a nitrogen atmosphere at 100 °C for a duration of 4 hours to eliminate potential contaminants and prevent the adsorption of undesirable gases. The Brunauer–Emmett–Teller (BET) method was employed to determine the specific surface area, while the pore size

distribution was evaluated using the Barrett–Joyner–Halenda (BJH) method. Morphological analysis of the synthesized catalyst was carried out using a Carl Zeiss Sigma 500 scanning electron microscope operating at 15 kV, which also facilitated the acquisition of elemental mapping images. Elemental composition and distribution within the composite were further examined through SEM-EDS spectroscopy. The reaction intermediates were characterized using high-resolution mass spectrometry (HRMS) employing a Waters XEVO G2-XS QTOF mass spectrometer coupled with ultra-performance liquid chromatography (UPLC). High-resolution transmission electron microscopy (HRTEM) was conducted using a JEOL JEM-2100 Plus instrument. Fourier-transform infrared (FTIR) spectra were recorded using a Bruker Alpha II spectrometer. Electrochemical impedance spectroscopy (EIS) was performed in the dark at 0.5 V (vs. Hg/HgO) using a Biologic VSP300 potentiostat over a frequency range of $10\text{-}10^5$ Hz. A three-electrode configuration was employed, consisting of the catalyst-loaded Ni foam as the working electrode, Hg/HgO as the reference electrode, and Pt as the counter electrode, with 1 M KOH as the electrolyte. The time-resolved studies were performed on DeltaFlex Modular Fluorescence Life-Time Spectrofluorimeter (HORIBA Scientific).

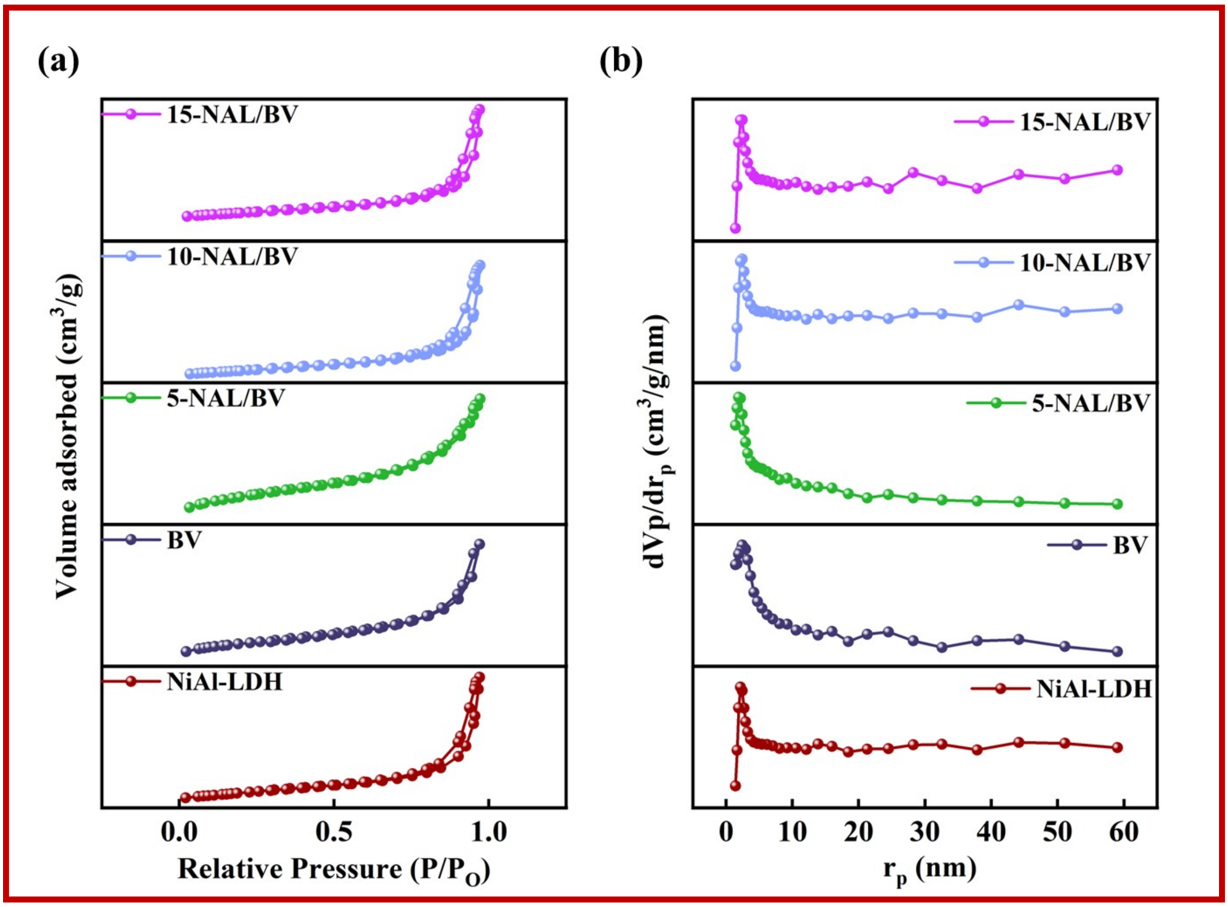

Fig. S1. (a) Nitrogen sorption isotherms, and (b) BJH plot of the synthesized photocatalysts.

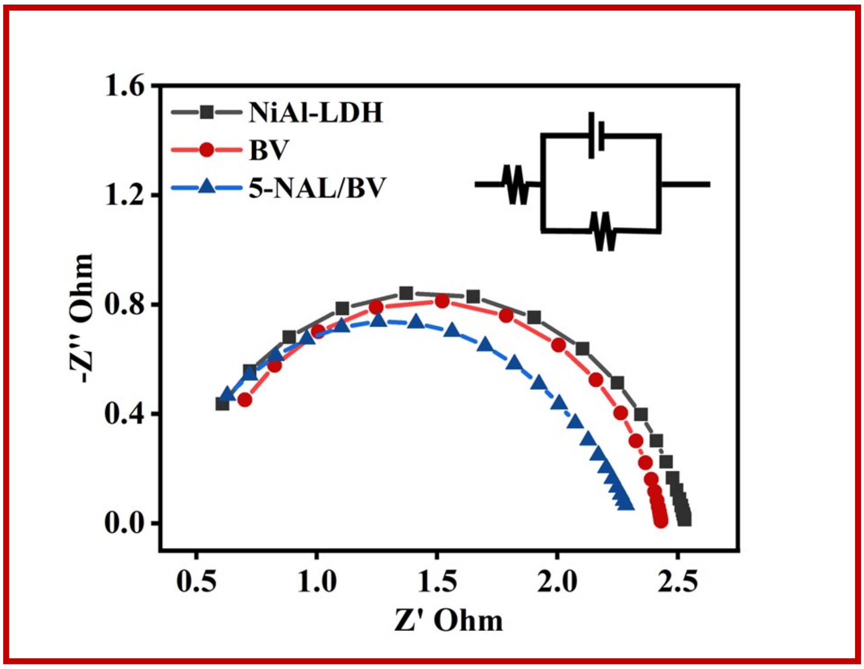

Fig. S2. Nyquist plots.

**Photocatalysts**	R_1	R_2	**Rct**
**NiAl-LDH**	0.6	2.52	1.92
**BV**	0.7	2.43	1.73
**5-NAL/BV**	0.62	2.28	1.66

Table S1. Comparison of R_1, R_2, and R_{ct} values obtained from EIS analysis of the synthesized materials.

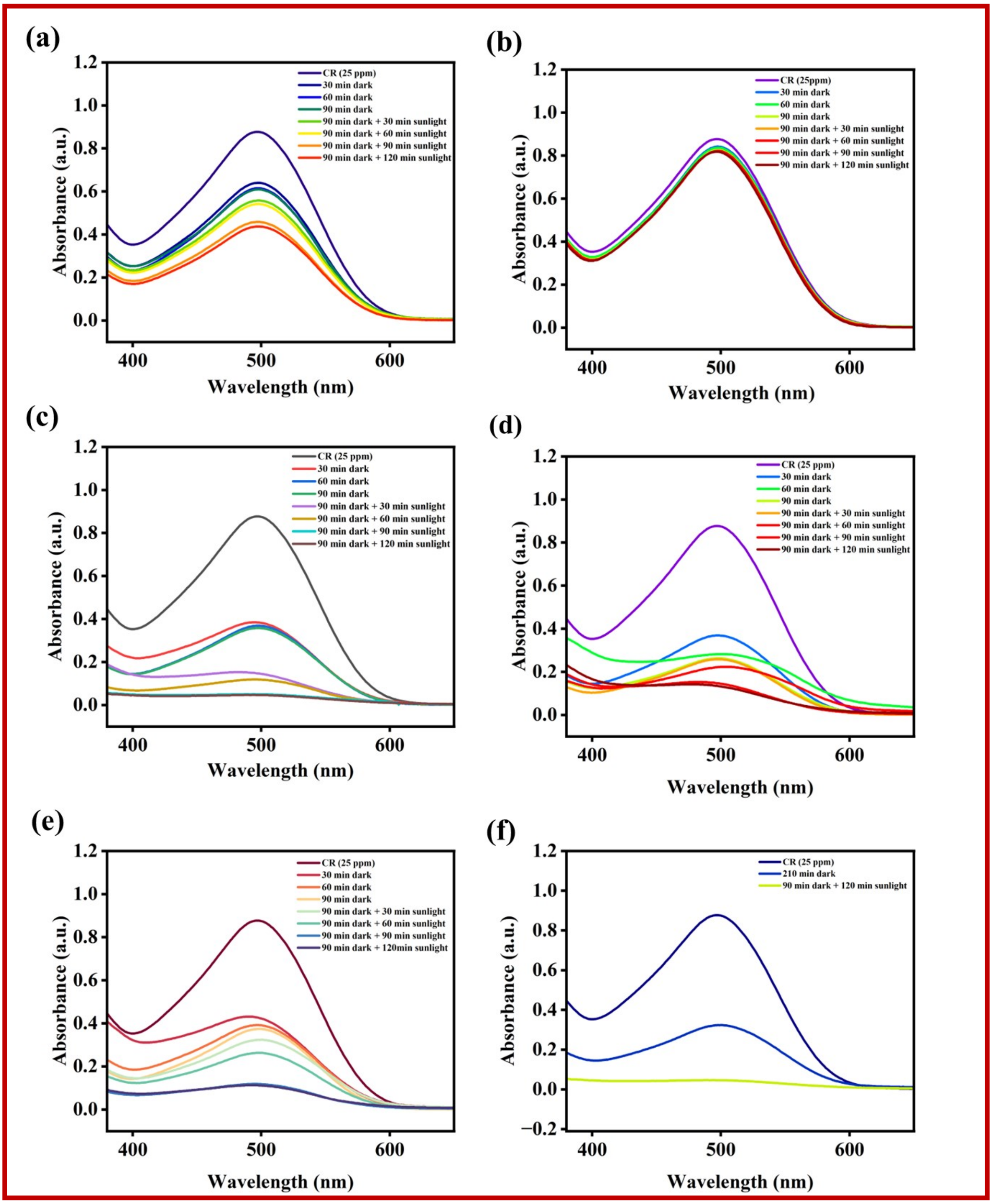

Fig. S3. Change in absorbance for (a) NiAl-LDH, (b) BV, (c) 5-NAL/BV, (d) 10-NAL/BV, (e) 15-NAL/BV, and (f) absorbance comparison of 5-NAL/BV photocatalyst under dark and illuminated conditions.

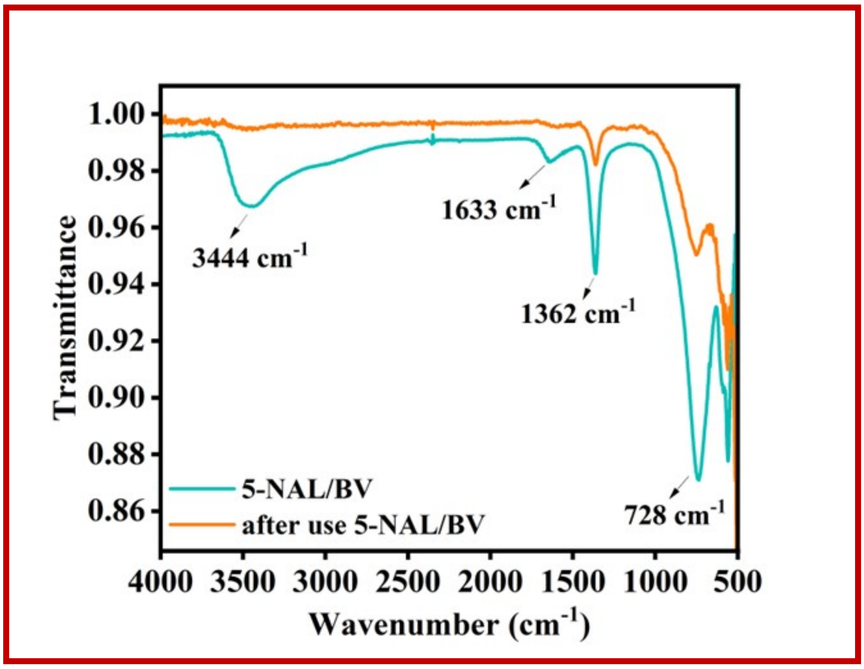

Fig. S4. FTIR spectra of 5-NAL/BV composite before and after degradation.

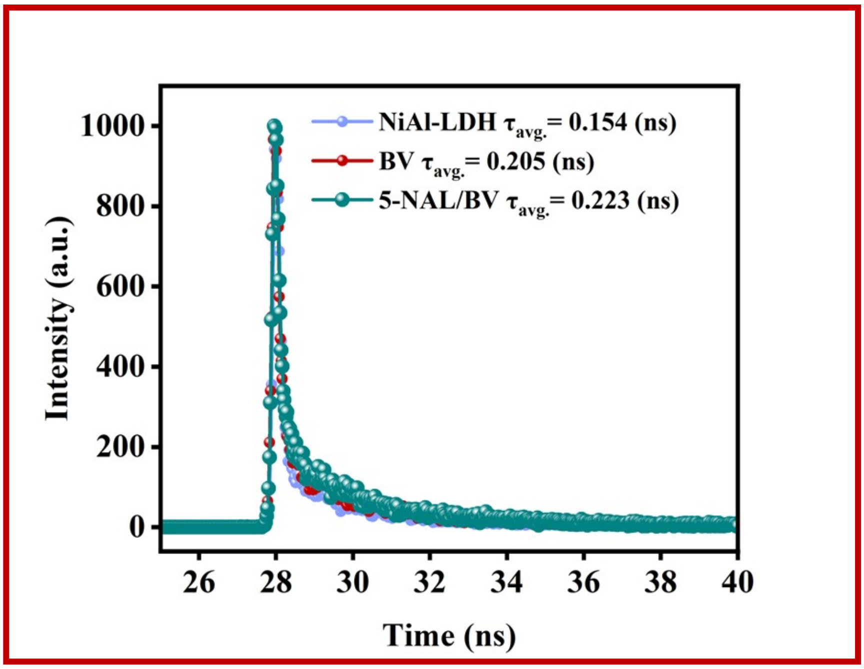

Fig. S5. TRPL decay profiles of the NiAl-LDH, BV, and 5-NAL/BV.

Sample	A_1	A_2	A_3	T_1	T_2	T_3	Chi sq.	$\tau_{avg.}$ (ns)
**NiAl-LDH**	163.66	30747.36	29790.04	2.3816	0.14765	0.14763	0.99894	0.154
**BV**	4643.73	3703.25	187.15	0.15413	0.15414	2.45879	0.98802	0.205
**5-NAL/BV**	4019.53	3690.55	215.67	0.1613	0.1613	2.43015	0.98833	0.223

Table S2. Fluorescence Lifetime Parameters of the NiAl-LDH, BV, and 5-NAL/BV.

(a)

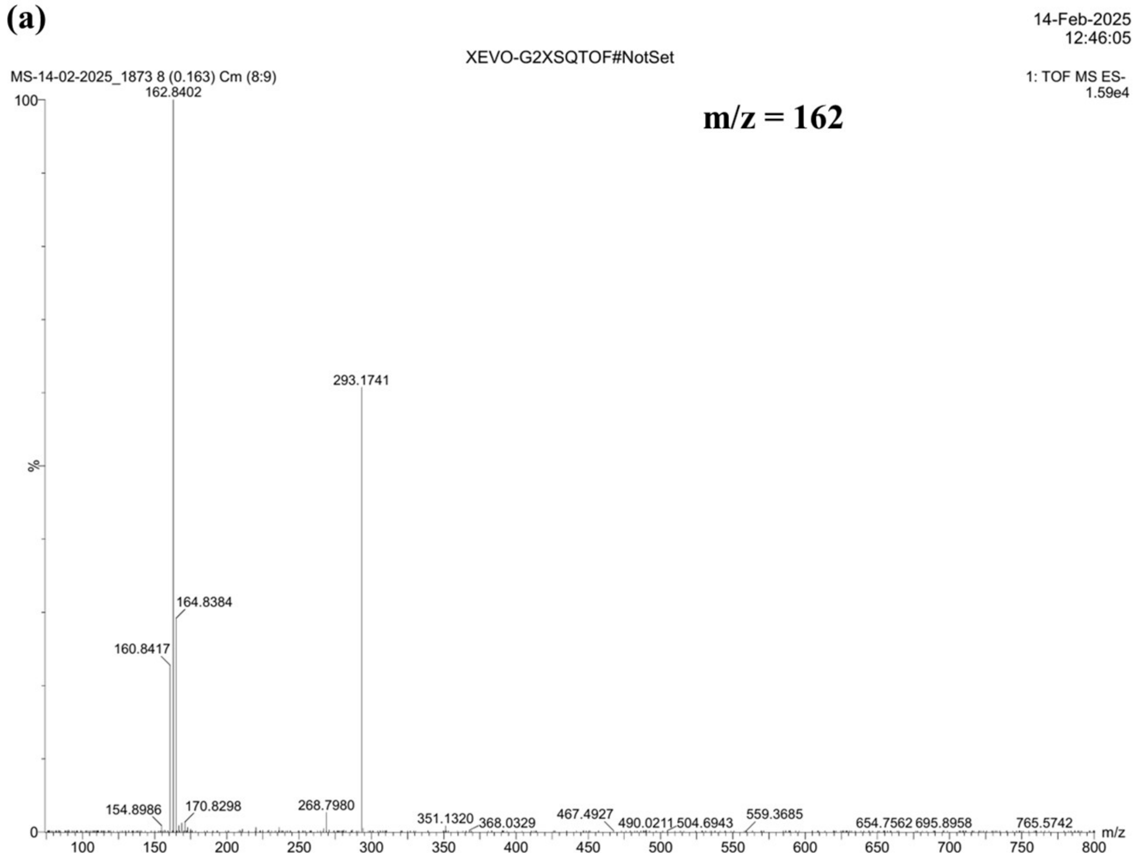
14-Feb-2025
12:46:05
XEVO-G2XSQTOF#NotSet
MS-14-02-2025_1873 8 (0.163) Cm (8:9)
1: TOF MS ES-
1.59e4
m/z = 162
162.8402
293.1741
164.8384
160.8417
154.8986
170.8298
268.7980
351.1320
368.0329
467.4927
490.0211
504.6943
559.3685
654.7562
695.8958
765.5742
%
m/z

(b)

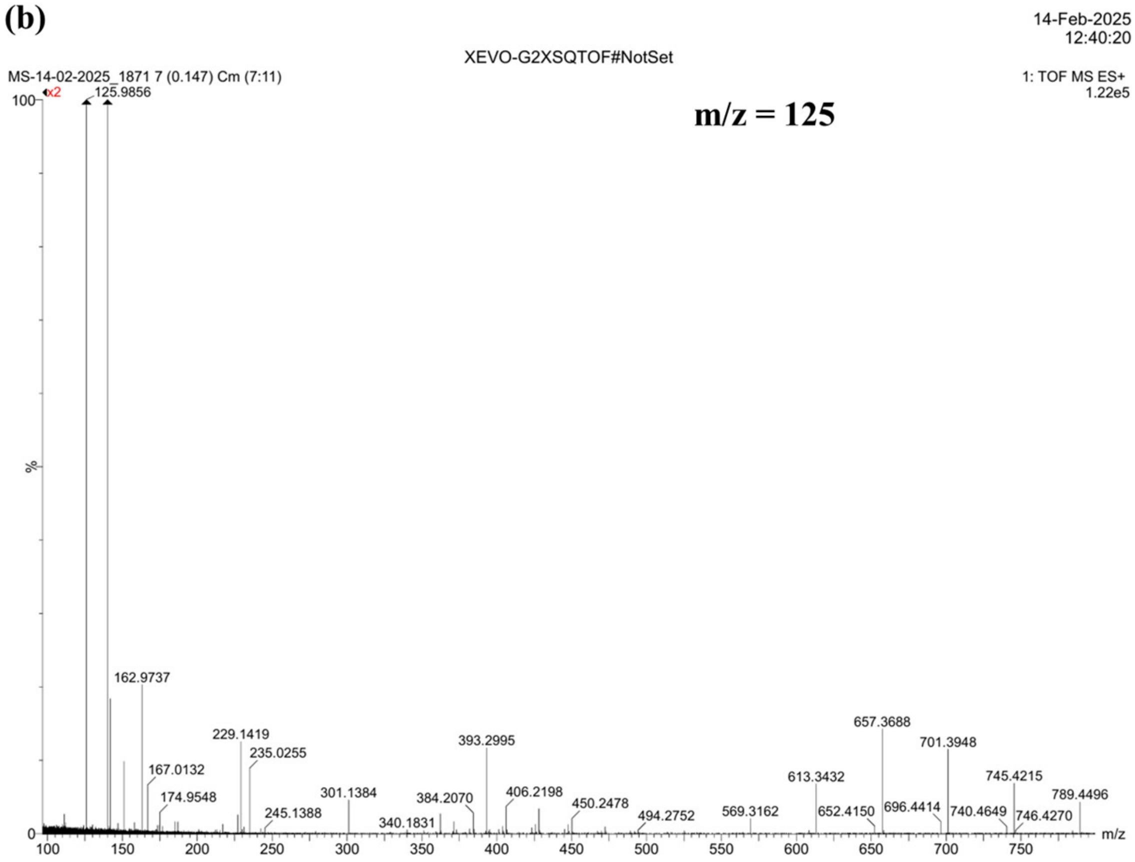
14-Feb-2025
12:40:20
XEVO-G2XSQTOF#NotSet
MS-14-02-2025_1871 7 (0.147) Cm (7:11)
1: TOF MS ES+
1.22e5
m/z = 125
x2
125.9856
162.9737
229.1419
235.0255
167.0132
174.9548
245.1388
301.1384
340.1831
384.2070
393.2995
406.2198
450.2478
494.2752
569.3162
613.3432
652.4150
657.3688
696.4414
701.3948
740.4649
745.4215
746.4270
789.4496
%
m/z

Fig. S6. HRMS spectrum of degraded CR solution (a) ES- mode, and (b) ES+ mode.
